# Structure–function analysis of the bacterial ClpE–ClpP AAA+ protease

**DOI:** 10.1016/j.jbc.2026.111403

**Published:** 2026-03-25

**Authors:** Mariasole De Rosa, Lisa Maag, Dirk Flemming, Irmgard Sinning, Marta Carroni, Axel Mogk

**Affiliations:** 1 Center for Molecular Biology of Heidelberg University (ZMBH), Heidelberg, Germany; 2 Heidelberg University Biochemistry Center (BZH), Heidelberg, Germany; 3 Science for Life Laboratory, Department of Biochemistry and Biophysics, Stockholm University, Stockholm, Sweden

**Keywords:** ATPase associated with diverse cellular activities, protein degradation, protein quality control

## Abstract

General and regulatory proteolysis in bacteria is executed by a set of ATP-dependent proteases composed of hexameric ring–forming AAA+ proteins and associated peptidase barrels (*e.g*., ClpP). These AAA+ proteases play crucial roles in stress protection and bacterial virulence. Here, we provide the first biochemical characterization of the potential drug target ClpE–ClpP from *Enterococcus faecalis*. We show that ClpE–ClpP forms an autonomous and efficient protease, which degrades misfolded and aggregated model substrates and the stress-responsive transcriptional regulator CtsR. This qualifies ClpE–ClpP as a central component of bacterial protein quality control systems and explains formerly reported stress-sensitive phenotypes of *clpE* mutants. ClpE substrate specificity is mediated by its N-terminal domain, which is crucial for targeting misfolded and aggregated proteins. ClpE assembles into a tetrahedral structure formed by four hexamers that interact *via* their coiled-coil M-domains. ClpP binding to ClpE tetrahedrons triggers the formation of large clusters of proteolytic complexes *in vitro* and *in vivo*. Such assembly in principle can allow for spatially confined proteolysis, separating the proteolytic activity of ClpE–ClpP complexes from other cellular processes. Indeed, ClpE M-domain mutants, which are deficient in cluster formation, exhibit increased toxicity *in vivo*.

General and regulatory proteolysis in bacteria is executed by ATP-dependent AAA+ proteases. These compartmentalized proteases consist of an ATPase component of the AAA+ protein family and a peptidase component. The AAA+ proteins form hexameric rings and thread target proteins into the associated peptidase barrel for degradation (1). Bacterial cells carry multiple AAA+ proteases that are composed of different AAA+ and peptidase components. The peptidase ClpP forms complexes with ClpA, ClpX, ClpC, or ClpE ATPases. AAA+ proteases have not only overlapping functions but also distinct functions and can differ in substrate specificity. Substrate selection is dictated by specific domains fused to the ATPase domains and by specific partner proteins (adaptors), which deliver bound substrates to the cognate AAA+ component (2).

AAA+ proteases play important roles in bacterial protein quality control (PQC) systems and virulence not only by degrading misfolded and aberrant proteins on one hand but also key regulatory proteins on the other. For instance, polypeptides, which originate from incomplete translation and are labeled with an SsrA-tag or polyalanine tails, are degraded by ClpX–ClpP (3, 4). ClpC–ClpP degrades misfolded and aggregated proteins (5, 6), and, accordingly, *clpC* mutant cells can exhibit heat-sensitive growth phenotypes (7, 8). AAA+ protease functions in PQC also involve proteolysis of transcriptional regulators that control stress responses. ClpX–ClpP degrades the global stress regulator Spx (9) and the starvation-specific sigma factor σ^S^ (10). The stress-responsive CtsR repressor is degraded by the ClpE–ClpP and ClpC–ClpP proteases *in vivo* (11, 12). Their central roles in bacterial proteostasis qualify AAA+ proteases as antibacterial drug targets (13, 14, 15).

The activities of AAA+ proteases in PQC are typically assisted by adaptor proteins. The ClpX-specific adaptors, SspB, YjbH, and RssB, specifically target SsrA-tagged polypeptides, Spx and σ^S^, respectively, for degradation (16, 17, 18). The degradation of misfolded and aggregated proteins by ClpC–ClpP requires the adaptor proteins, MecA and McsA/McsB, respectively (19, 20). These adaptors do not only provide target specificity but also are additionally required for activation of ClpC ATPase activity (19, 21). ClpC is kept inactive in the absence of adaptor proteins by forming a nonhexameric resting state, which displays low ATPase activity and cannot associate with ClpP (22). Adaptors convert the inactive resting state into an active hexameric assembly (22). This activity control of ClpC is crucial for the viability of bacteria, as constitutive ClpC activation by mutation (22) or natural cyclic peptides (23, 24, 25) causes lethal uncontrolled proteolysis.

Next to the major ClpX–ClpP and ClpC–ClpP proteases are selective Gram-positive bacteria, including *Bacillus subtilis* and the pathogens *Listeria monocytogenes*, *Streptococcus pneumonia*, and *Enterococcus faecalis* (*Ef*), that harbor ClpE–ClpP as an additional proteolytic system. ClpE is composed of two AAA domains (AAA1, AAA2) and a coiled-coil middle (M-) domain (MD), which shares sequence homology with the ClpC MD. Like ClpX and ClpC, it carries the ClpP-interaction motif “VGF” in the AAA2 domain. ClpE differs from ClpC in its N-terminal domain (NTD), which shares sequence homology with the first NTD of the disaggregase ClpG (26).

*In vivo* analysis links ClpE–ClpP activity to bacterial PQC. The *clpE* gene is typically organized as a monocistronic operon, and its expression is strongly induced by stress conditions, including high temperatures (27) and low pH (28, 29, 30). Furthermore, exposure to antibacterial compounds induces expression of *L. monocytogenes* and *Ef clpE* (31, 32). Control of *clpE* expression mostly involves the stress-responsive transcriptional repressor CtsR (27, 33). Enhanced expression of ClpC–ClpP and ClpE–ClpP proteases in *Ef ctsR* knockout cells attenuates virulence in an invertebrate model (34), documenting a need to tightly control protease production. Notably, *B. subtilis* ClpE only transiently accumulates upon heat shock, as it is rapidly degraded in a ClpC–ClpP-dependent manner (12, 35). This finding hints at a need to limit ClpE–ClpP activity to immediate stress conditions.

In agreement with its stress-dependent expression, ClpE has been implicated in the processing of misfolded and aggregated proteins. *Lactococcus lactis* ClpE–ClpP contributes to the degradation of aberrant (*e.g*., truncated) proteins, and *clpE* mutant cells show increased sensitivity to puromycin treatment (36). *B. subtilis* ClpE localizes to protein aggregates, and *clpE* knockout cells exhibit a delay in the removal of protein aggregates (6, 12). A role of *B. subtilis* ClpE in PQC is further supported by a delayed attenuation of stress-induced *clpC* and *clpP* gene expression in *clpE* mutant cells, which is likely caused by a delay in the removal of stress-induced protein damage (12, 37). Furthermore, *Streptococcus mutans* and *S. pneumoniae clpE* knockout strains are more sensitive to high temperatures (38, 39). The stress-sensitive phenotype of *S. pneumoniae clpE* mutant cells correlates with a strongly reduced virulence in a mouse infection model (40), pointing to a relevance of ClpE function in PQC during host infection.

A biochemical characterization of the ClpE–ClpP protease has not been reported to date, and a reconstitution of proteolytic activities toward misfolded and aggregated proteins is lacking. Furthermore, it is unclear whether ClpE functions autonomously or requires adaptor proteins for substrate targeting or activation. This point is relevant as ClpE and ClpC share a similar domain organization, including a coiled-coil MD, that is crucial for keeping ClpC inactive in the absence of adaptors (22). Here, we report on the first *in vitro* characterization of ClpE–ClpP. We show that ClpE–ClpP can efficiently and autonomously degrade misfolded and aggregated model substrates and the transcriptional regulator CtsR. ClpE activity is modulated by its NTD and MD in a substrate-specific manner. The interplay of both domains also controls ClpE activity *in vivo* and its assembly into large complexes. Our findings describe the basic mechanism of ClpE–ClpP activity and provide valuable information for potential future drug development.

## Results

### ClpE–ClpP autonomously degrades diverse PQC substrates

**Figure 1:**
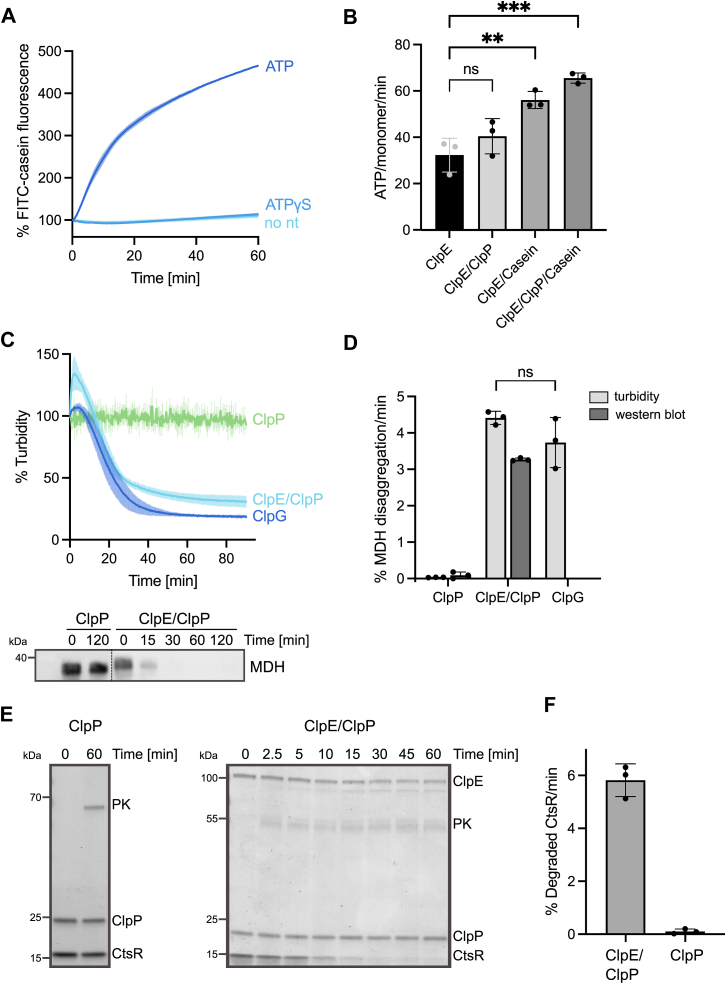
**ClpE–*Sa* ClpP autonomously degrades diverse protein quality control substrates.***A*, FITC–casein degradation by ClpE–*Sa* ClpP in the absence (no nt) and the presence of nucleotides was followed by monitoring the increase in FITC–casein fluorescence. Initial FITC–casein fluorescence was set as 100%. *B*, ATPase activity of ClpE was determined in the absence and presence of *Sa* ClpP and casein as indicated. *C*, disaggregation of aggregated malate dehydrogenase (MDH) was monitored by turbidity measurements in the presence of indicated components. The initial turbidity of MDH aggregates was set as 100%. MDH disaggregation was additionally monitored by Western blot analysis using MDH-specific antibodies, documenting degradation of MDH aggregates by ClpE–*Sa* ClpP. *D*, MDH disaggregation rates were determined based on turbidity measurements (% decrease MDH turbidity/min) or Western blot analysis (% degraded MDH/min). *E*, degradation of CtsR was analyzed at indicated time points by SDS-PAGE analysis and Coomassie staining. Protein identities are indicated (PK, pyruvate kinase). *F*, CtsR degradation rates were determined based on SDS-PAGE analysis. Error bars show standard deviations (n = 3). Statistical analysis was performed either by one-way ANOVA with Dunnett’s multiple comparison test (*B*) or by an unpaired *t* test with Welch’s correction (*D*). ns, not significant, ∗∗*p* < 0.01, and ∗∗∗*p* < 0.001. Sa, Staphylococcus aureus.

We purified ClpE from the pathogen *Ef* and first tested for adaptor-independent, autonomous activity. Due to the stress-dependent expression of *clpE*, we first probed for proteolytic activity toward the disordered substrate casein as a mimic for soluble, misfolded proteins. Here, we used fluorescently labeled casein (FITC–casein), whose degradation is monitored by an increase in fluorescence intensity. We used *Staphylococcus aureus* (*Sa*) ClpP as a peptidase partner, as we observed the formation of functional complexes between *Mycobacterium tuberculosis* ClpC1 and *Sa* ClpP before, indicating species-independent cooperation between the AAA+ partner and the peptidase (24). Since expression of *clpE* is frequently upregulated by low pH, we additionally created a pH profile of ClpE–*Sa* ClpP proteolytic activity. FITC–casein was degraded by ClpE–*Sa* ClpP, demonstrating that ClpE, in principle, does not require partner proteins for activity (Fig. 1*A*). The highest activities were observed for pH values between 6 and 7, whereas a substantial decline was observed below (pH 5.5) or above (pH 7.5) these values (Fig. S1*A*). A similar profile was obtained not only when determining ClpE ATPase activity (Fig. S1, *B* and *C*) but also for *Sa* ClpP, whose peptidase activity was even more affected by lowering pH. We therefore performed degradation and ATPase assays throughout this study at pH 6.5. FITC–casein degradation by ClpE–*Sa* ClpP was dependent on ATP hydrolysis, as no proteolysis was observed in the absence of nucleotide or in the presence of nonhydrolyzable ATPγS (Figs. 1*A* and S1*D*).

We compared the FITC–casein degradation activity of ClpE–*Sa* ClpP to the one of adaptor-activated *Sa* ClpC–*Sa* ClpP. ClpE–*Sa* ClpP activity was comparable to MecA-activated ClpC–*Sa* ClpP and 1.8-fold higher as compared with McsA–McsB-activated ClpC–*Sa* ClpP (Fig. S2*A*). This underlines that ClpE–*Sa* ClpP does not require adaptor proteins for high proteolytic activity, unlike ClpC–ClpP.

We next analyzed the impact of *Sa* ClpP and substrate FITC–casein on ClpE ATPase activity (Fig. 1*B*). Basal ATPase activity of ClpE (32 ATP/monomer/min) was enhanced by *Sa* ClpP and casein (1.25- and 1.75-fold, respectively). The presence of both *Sa* ClpP and casein further increased ClpE ATPase activity, though the effect was not synergistic but additive (Fig. 1*B*). We infer that the *Sa* ClpP association and particularly substrate binding stimulates ClpE ATPase activity.

We then probed for ClpE–*Sa* ClpP activity toward protein aggregates since ClpE has been implicated in protein disaggregation *in vivo* (12). We used aggregated malate dehydrogenase (MDH) as an established model substrate and monitored disaggregation directly by turbidity measurements (Fig. 1, *C* and *D*). Addition of ClpE–*Sa* ClpP to MDH aggregates caused an initial increase in sample turbidity, which was followed by a rapid and pronounced decrease, indicating efficient MDH disaggregation. The kinetics of MDH disaggregation by ClpE–*Sa* ClpP were comparable to the standalone disaggregase *Pseudomonas aeruginosa* ClpG (41) that we used as a reference (Fig. 1, *C* and *D*), demonstrating the high disaggregation potential of ClpE–*Sa* ClpP. We confirmed ClpE–*Sa* ClpP disaggregation activity by additionally demonstrating degradation of MDH aggregates *via* Western blotting (Fig. 1*C*). Kinetics of MDH turbidity decrease and MDH degradation by ClpE–*Sa* ClpP were similar (4.4 *versus* 3.3% MDH/min, respectively) (Fig. 1*D*). Notably, ClpE–*Sa* ClpP–mediated degradation of aggregated MDH was approximately twofold faster as compared with *Sa* McsA–McsB-activated ClpC–*Sa* ClpP, which is implicated in protein disaggregation in *B. subtilis* (5, 20) (Fig. S2*B*).

We also monitored refolding of aggregated MDH by ClpE in the absence of *Sa* ClpP and observed efficient MDH reactivation (Fig. S2, *C–E*). MDH refolding yields determined in the presence of ClpE were similar to those determined in the presence of ClpG, underlining the high disaggregation potential of ClpE.

We then probed for ClpE–*Sa* ClpP activity toward *B. subtilis* CtsR, the stress-response transcriptional repressor downregulating the expression of *clpC*, *clpE*, and *clpP* genes in Gram-positive bacteria (42, 43). ClpE contributes to CtsR degradation *in vivo* (12), and accordingly, we noticed rapid ATP-dependent degradation of *B. subtilis* CtsR by ClpE–*Sa* ClpP *in vitro* (Fig. 1, *E* and *F*).

Together, our findings demonstrate that ClpE targets soluble misfolded and aggregated PQC substrates and the stress-responsive CtsR regulator. These findings support a crucial function of ClpE–ClpP in bacterial PQC systems.

### Characterization of the *Ef* ClpE–ClpP complex

**Figure 2:**
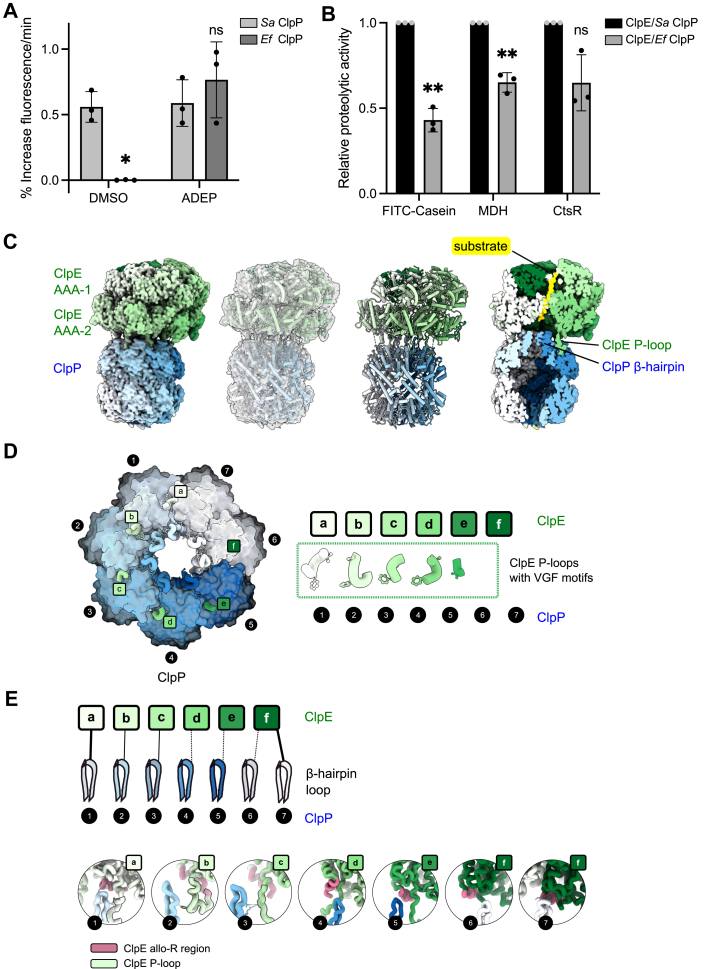
**Characterization of ClpE–*Ef* ClpP complexes.***A*, degradation of the fluorescent peptide substrate LY-AMC by *Sa* or *Ef* ClpP was monitored in the absence or the presence of ADEP. LY-AMC degradation rates were calculated. *B*, degradation of FITC–casein, aggregated MDH, and CtsR by ClpE complexed with either *Sa* or *Ef* ClpP. Respective degradation rates were calculated, and the activity of ClpE–*Sa* ClpP for each substrate was set to 1. Error bars show standard deviations (n = 3). Statistical analysis was performed either by an unpaired *t* test with Welch’s correction (*A*) or by one-way ANOVA with Dunnett’s multiple comparison test (*B*). ns, not significant, ∗*p* < 0.05, and ∗∗*p* < 0.01. *C*, cryo-EM structure of the *Ef* ClpE–ClpP complex with the fitted model inside. ClpE subunits are colored in different shades of *green* and ClpP subunits in different shades of *blue*. The trapped substrate FITC–casein inside the ClpE channel is shown in *yellow* in a cut-through view of the map. Complex formation involves ClpE P-loop and ClpP β-hairpin loops. *D*, interaction between the ClpE ring and ClpP is mainly mediated *via* the P-loop VGF motifs, displayed with a *licorice cartoon representation*, anchored onto the ClpP H-site external surface (*left panel*). ClpP β-hairpins also displayed as *licorice cartoons*, also contribute to the ClpE–ClpP interaction. Interaction *via* the P-loops VGF motifs is tighter for ClpE subunits a to d, whereas the seam subunit e does not make contact (*right panel*). *E*, contacts between ClpP N-terminal β-hairpins and ClpE. The strength of the interaction is indicated by *line thickness* (*top panel*). Interaction details between ClpP β-hairpins and the ClpE subunits are shown in the *bottom row*. The allo-R regions are shown in *light magenta*, extended P-loops are shown in *light green* with a *dark silhouette*, and β-hairpins are shown in *blue/white*. The tightest contacts are made at the interface between the seam subunit f and subunit a. Ef, Enterococcus faecalis; MDH, malate dehydrogenase; Sa*,* Staphylococcus aureus.

We next reconstituted the authentic *Ef* ClpE–ClpP protease. *Sa* and *Ef* ClpP exhibit high sequence and structural similarity (Fig. S3), yet we observed differences between the autonomous hydrolytic activities of the peptidases. *Sa* ClpP autonomously degraded the fluorescently labeled peptide LY-AMC, whereas *Ef* ClpP required activation by the antibiotic ADEP, which mimics binding of an AAA+ partner protein (Figs. 2*A* and S3*B*). This implies that *Ef* ClpP predominantly exists in a compressed conformation and requires binding of the AAA+ partner or ADEP for transition to the extended, active conformation. We noticed some sequence alterations between the two peptidases that might be causative for this difference. The handle region, which controls the switch between active extended and inactive compressed conformations, includes two stronger sequence alterations between *Sa* and *Ef* ClpP (K145D, R152S) (Fig. S3*A*). Another difference was found in the core domain close to the catalytic Ser98 residue: C91V (*Sa*–*Ef* ClpP). This residue is located close to the H-site, which interacts with the P-loop of the cooperating AAA+ partner and is involved in allosteric communication within ClpP.

ClpE–*Ef* ClpP complexes degraded all tested substrates (FITC–casein, aggregated MDH, and *B. subtilis* CtsR), and degradation kinetics were partially reduced by 1.5- to 2.4-fold as compared with ClpE–*Sa* ClpP (Figs. 2*B* and S3, *C–E*). This documents autonomous and efficient proteolysis by authentic ClpE–*Ef* ClpP.

We next determined the cryo-EM structure of ClpE–*Ef* ClpP complexes. Here, we used the ClpE–E373A mutant as it forms canonical protease complexes (see below), facilitating structure determination. The *Ef* ClpE–ClpP complex was assembled in the presence of the model substrate FITC–casein, and the structure was resolved at an overall resolution of 2.88 Å (Fig. S4). Extensive global and focused 3D classification was performed to identify potential alternative conformational states. Nevertheless, all classes converged to a single dominant conformation at medium-to-high resolution (3.2–2.9 Å) (Fig. S4). Local refinement of the *Ef* ClpP barrel yielded a highly D7 symmetric reconstruction at 2.49 Å resolution (Fig. S4), closely resembling *Sa* ClpP in the MecA–ClpC–ClpP complex, with catalytic residues precisely aligned (Fig. S5*A*).

The *Ef* ClpE–ClpP structure recapitulates the canonical architecture previously observed for ClpC–ClpP and ClpA–ClpP complexes (44, 45) with the hexameric AAA+ ClpE ring docked onto the ClpP peptidase barrel (Figs. 2*C* and S5*B*). The NTDs and MDs of ClpE are not resolved in the cryo-EM reconstruction, indicating pronounced conformational flexibility of these regions. The substrate FITC–casein is visible within the central channel of ClpE, yet its binding did not result in stabilization of the NTDs or MDs (Fig. 2*C*).

Interactions between ClpE and *Ef* ClpP are highly similar to those described before between ClpC and *Sa* ClpP (Figs. 2, *C–E* and S5*B*) (44). ClpE P-loops containing the conserved VGF motif engage the ClpP hydrophobic H-sites (Fig. 2, *C* and *D*). As observed for ClpC–ClpP, this interaction interface is asymmetric with the strongest interactions for ClpE subunits a–d, whereas no contact is observed for the seam subunit f (Fig. 2*D*). The N-terminal β-hairpins of *Ef* ClpP establish additional contacts with the ClpE AAA2 domain. Specifically, these interactions involve a region (V615–F623) adjacent to the α-helix harboring the AAA2 R-finger, previously noticed for the *Sa* ClpC–ClpP complex and termed allo-R (Fig. 2*E*) (44). We additionally observed contacts with a portion of the extended ClpE P-loop (G594–S611) (Fig. 2*E*). As observed for *Sa* ClpC–ClpP, the β-hairpin interaction interface is also asymmetric, with tight contacts to the allo-R segments of ClpE subunits a and f, which are positioned at the bottom of the ClpE hexamer (Fig. 2*E*).

Within the complex, the ClpE ring aligns with the *Sa* ClpC ring on one side, whereas the seam subunits and their adjacent protomer are displaced upward (Fig. S5*B*), potentially reflecting conformational dynamics associated with substrate threading. The ATP-binding pockets of ClpE are clearly resolved, and the AAA1 ring has ATP bound at all subunits, whereas the AAA2 ring has ATP bound everywhere but at the two seam subunits, e and f (Fig. S5*C*). The R-finger from the neighboring subunit is always within 2 to 4 Å distance, thus indicating that all the ATP-bound subunits are competent for hydrolysis. This activity pattern of AAA domains is in parts different from the one observed for *Sa* ClpC in a complex with ClpP, yet both structures represent single snapshots of the respective ATPase and threading cycles, explaining such differences. Notably, ClpE AAA1 and AAA2 subunits are in parts in different activity states and offset with AAA2 seam subunits being inactive, and these features are shared with *Sa* ClpC.

Collectively, these findings establish that ClpE engages *Ef* ClpP through canonical interaction principles conserved among AAA+ Clp ATPases. The absence of substantial differences between *Ef* ClpE–ClpP and *Sa* ClpC–ClpP interactions supports functional interchangeability of the peptidases. We used *Sa* ClpP for subsequent mechanistic analyses, with *Ef* ClpP included to validate key observations.

### Role of individual AAA domains for ClpE activity

**Figure 3:**
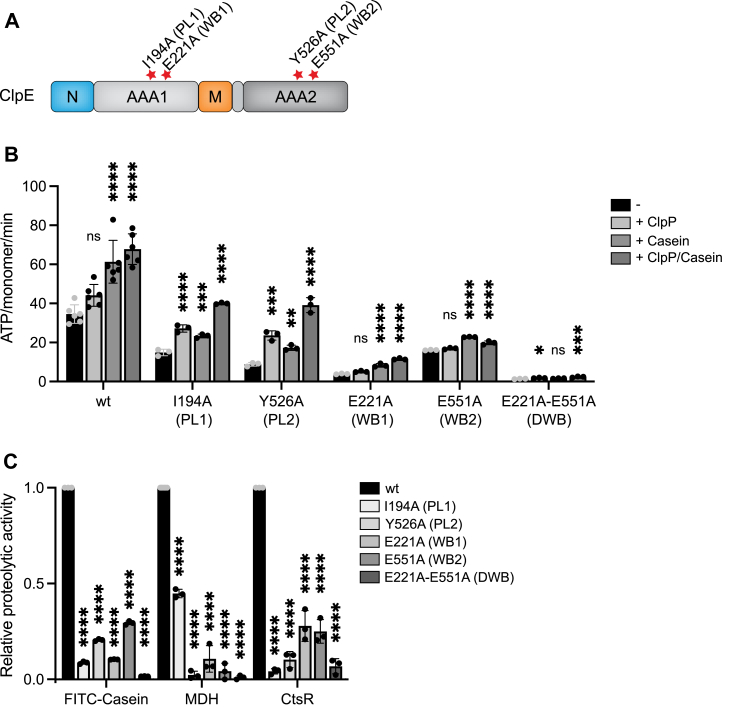
**Role of individual AAA1/2 domains for ClpE activity.***A*, domain organization of ClpE. ClpE consists of two AAA domains (AAA1, AAA2), an N-terminal domain (N), and a middle domain (M). Positions of Walker B (E221A, E551A) and pore loop (I194A, Y526A) mutations are indicated. *B*, ATPase activities of ClpE wt, Walker B (WB1, WB2, and DWB), and pore loop (PL1, PL2) mutants were determined in the absence and presence of *Sa* ClpP and casein. *C*, relative proteolytic activities of ClpE Walker B and pore loop mutants were determined for the substrates FITC–casein, aggregated MDH, and CtsR in the presence of *Sa* ClpP. Degradation rates were determined based on changes (%/min) in FITC–casein fluorescence, MDH levels were determined by Western blot analysis, and CtsR levels were determined by SDS-PAGE analysis. The proteolytic activity of ClpE wt was set to 1 for each substrate, and the relative proteolytic activities of ClpE mutants are shown. Error bars show standard deviations (n ≥ 3). Statistical analysis was performed by one-way ANOVA with Dunnett’s multiple comparison test (*B* and *C*). ns, not significant, ∗*p* < 0.05, ∗∗*p* < 0.01, ∗∗∗*p* < 0.001, and ∗∗∗∗*p* < 0.0001. MDH, malate dehydrogenase; *Sa*, *Staphylococcus aureus.*

ClpE harbors two AAA domains (AAA1, AAA2). We dissected their individual contributions to ClpE activity by mutating the conserved WalkerB motifs (E221A, E551A) crucial for ATP hydrolysis, and the pore loop residues (I194A, Y526A), crucial for substrate threading (Fig. 3*A*). Basal ATPase activities of pore loop mutants were reduced as compared with ClpE wt and enhanced by substrate casein and ClpP (Fig. 3*B*). ClpE single Walker B mutants exhibited reduced ATPase activities, whereas ClpE–E221A–E551A (ClpE–DWB) was inactive (Fig. 3*B*). Thus, both AAA domains contribute to ClpE ATPase activity, and ATP hydrolysis in both rings is required for high basal ATPase activity. ATPase activity of ClpE–E221A (WB1) was increased by *Sa* ClpP and casein, yet the activity remained low. Notably, *Sa* ClpP did not enhance ATP hydrolysis of ClpE–E551A (WB2), suggesting that *Sa* ClpP specifically enhances ATPase activity of the AAA2 ring (Fig. 3*B*). This can be explained by the association of *Sa* ClpP with the AAA2 ring.

We next probed for the role of individual AAA domains in substrate degradation and tested the proteolytic activities of respective mutants toward FITC–casein, aggregated MDH, and *B. subtilis* CtsR (Fig. 3*C*). All mutants displayed strongly reduced degradation activities toward all tested substrates. No proteolytic activity was determined for ClpE–DWB, confirming that substrate degradation requires ATP hydrolysis (Fig. 3*C*). The specific defects of the tested mutants were in parts substrate dependent. ClpE–I194A (PL1) retained substantial activity toward aggregated MDH, whereas it hardly degraded CtsR (Fig. 3*C*). Contrary, ClpE–E221A and ClpE–E551A (WB1, WB2) were deficient in MDH disaggregation while retaining partial activity toward FITC–casein and CtsR. We speculate that such differences are caused by diverse modes of substrate recognition and differences in substrate stabilities. We conclude that the integrity of both AAA domains is required for high ClpE–*Sa* ClpP proteolytic activities (also summarized in Table S2).

### ClpE hexamers interact *via* MDs to form tetrahedral assemblies

**Figure 4:**
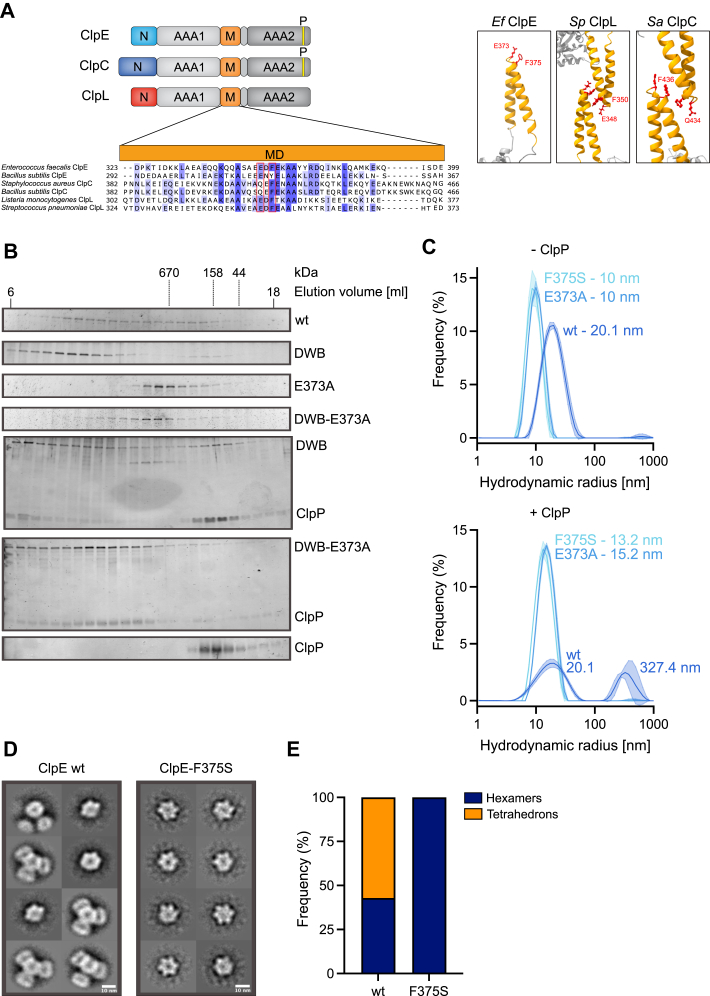
**ClpE forms MD-dependent tetrahedrons.***A*, domain organizations of ClpE, ClpC, and ClpL and sequence alignment of the MD of ClpE, ClpC, and ClpL proteins from the indicated species. Residues that are crucial for formation of head-to-head MD interactions are boxed in *red*, and their interactions are shown for the *Streptococcus pneumoniae* ClpL dimer and for the *Staphylococcus aureus* ClpC resting state. An AlphaFold2 model of the ClpE MD is shown. *B*, oligomeric states of ClpE wt, ClpE–E221A–E551A (DWB), and the MD mutants, ClpE–E373A and ClpE–E221A–E551A–E373A, were determined in the absence and presence of *Sa* ClpP by Superose 6 size-exclusion chromatography (SEC). Elution fractions were analyzed by SDS-PAGE and silver staining. *C*, particle size distributions (% frequency) of DLS data were determined for ClpE wt and the MD mutants, E373A and F375S, in the absence (*top*) and presence (*bottom*) of *Sa* ClpP. The hydrodynamic radius of the most populated state is indicated. Standard deviations (n = 30) are shown as *shaded areas*. *D*, 2D class averages of ClpE wt and ClpE–F375S based on negative-stain EM. The scale bar represents 10 nm. *E*, populations (%) of diverse ClpE assembly states (tetrahedrons and hexamers) based on 2D class averages were determined for ClpE wt and ClpE–F375S. Evaluated particles: n_wt_ = 7247, n_F375S_ = 6941. DLS, dynamic light scattering; MD, middle domain; *Sa*, Staphylococcus aureus.

Bacterial AAA+ proteins, which harbor an MD consisting of a single coiled-coil wing, can form alternative assembly states mediated by head-to-head MD–MD interactions. Those assemblies either represent two interacting AAA+ half-spirals (inactive ClpC resting state) (22) or dimers and tetramers of ClpC or ClpL AAA+ rings (Fig. S6*A*) (23, 46, 47, 48). We determined the oligomeric state of ClpE–wt by size-exclusion chromatography (SEC), dynamic light scattering (DLS), and glutaraldehyde (GA) crosslinking. To probe for a role of the MD in ClpE oligomerization, we mutated the conserved MD residues, E373 and F375. The homologous residues of the ClpC and ClpL MD are crucial for MD–MD interactions and the formation of larger ClpC and ClpL assemblies (Fig. 4*A*) (22, 47, 48). To stabilize potential ClpE assemblies, SEC runs were performed in the presence of ATP, and ATPase-deficient ClpE–DWB was included to ensure maximal complex integrity. ClpE–wt and ClpE–DWB formed large complexes in SEC runs that eluted prior to a 670 kDa protein standard, suggesting the formation of assemblies larger than single hexameric rings (Fig. 4*B*).

Large ClpE–wt complexes were also determined in the presence of ATPγS (hydrodynamic radius of 20.1 nm) in DLS measurements (Figs. 4*C* and S6*B*), and crosslinked ClpE–wt formed high-molecular complexes that hardly migrated into the SDS-gel (Fig. S6*C*). The formation of large complexes was dependent on the MD, as both ClpE MD mutants (E373A, F375S) formed smaller complexes in SEC and DLS (hydrodynamic radius of 10 nm) with particle sizes similar to a hexameric ring (Figs. 4, *B* and *C* and S6*B*). These findings were confirmed by GA crosslinking, as both MD mutants were predominantly crosslinked to a hexameric species, as validated by a crosslinked ClpB hexamer that served as a reference (Fig. S6*C*).

Both ClpE–WT and ClpE–E373A assemblies are associated with *Sa* ClpP, yet the consequences on complex sizes strongly differed. ClpE–DWB–*Sa* ClpP complexes were very large and eluted in or close to the void volume of the Superose 6 SEC column (Fig. 4*B*). Similarly, DLS measurements revealed the formation of large and heterogenous complexes of ClpE–wt and *Sa* ClpP (radii of 187.4–572.2 nm, most populated radius of 327.4 nm) (Figs. 4*C* and S6*B*). In contrast, ClpE–DWB–E373A–*Sa* ClpP complexes eluted much later in Superose 6 SEC runs (Fig. 4*B*), and ClpE–E373A and ClpE–F375S–*Sa* ClpP complexes were much smaller and well defined in DLS measurements (mean radius of 13.2–15.2 nm) (Figs. 4*C* and S6*B*). We determined the same assembly sizes when studying the complex formation of ClpE–wt and MD mutants with *Ef* ClpP, confirming the interchangeability of both peptidases (Fig. S6*D*).

To determine the structural organizations of ClpE–wt and ClpE–E373A–ClpE–F375S assemblies, we performed negative-staining EM. ClpE–wt formed single hexamers and a tetrahedron-like structure composed of four hexamers (Figs. 4, *D* and *E* and S6*E*). This assembly state has been described before for ClpC assemblies bound to an activating pArg substrate or the antibacterial peptide cyclomarin A (23, 46) and ClpL (48). The tetrahedron structure explains the formation of large and heterogenous complexes with ClpP, as one tetrahedron offers four binding sites for ClpP, and each ClpP oligomer in turn can interact with two tetrahedrons, leading to the formation of a large meshwork. The four ClpE hexamers interact *via* their MDs, and accordingly, the ClpE–F375S MD mutant formed exclusively single hexameric rings (Figs. 4, *D* and *E* and S6*F*). We infer that ClpE forms large, hexamer-based assemblies *via* MD interactions.

**Figure 5:**
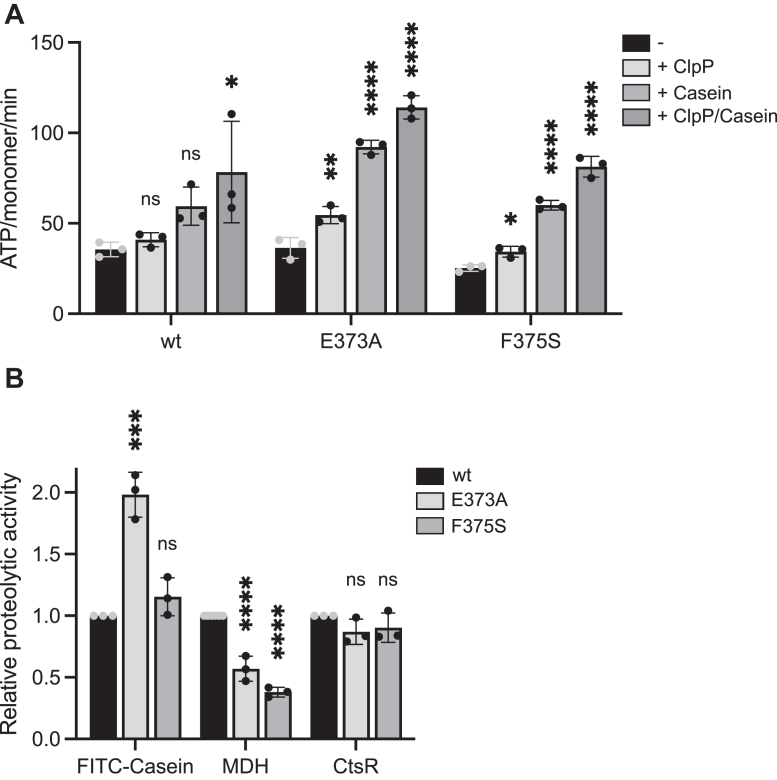
**ATPase and proteolytic activities of ClpE MD mutants.***A*, absolute ATPase activities of ClpE wt and MD mutants, E373A and F375S, were determined in the absence and presence of *Sa* ClpP and casein. n = 3. *B*, relative proteolytic activities of ClpE MD mutants were determined for the substrates FITC–casein, aggregated MDH, and CtsR in the presence of *Sa* ClpP. Degradation rates were determined based on changes (%/min) in FITC–casein fluorescence, MDH turbidity, and by SDS-PAGE analysis (CtsR). The proteolytic activity of ClpE wt was set to 1 for each substrate, and the relative proteolytic activities of ClpE mutants are shown. Error bars show standard deviations (n = 3). Statistical analysis was performed by one-way ANOVA with Dunnett’s multiple comparison test (*A* and *B*). ns, not significant, ∗*p* < 0.05, ∗∗*p* < 0.01, ∗∗∗*p* < 0.001, and ∗∗∗∗*p* < 0.0001. MD, middle domain; MDH, malate dehydrogenase; *Sa*, Staphylococcus aureus.

We next analyzed the impact of the diverse ClpE assembly states on protein activity by comparing ATPase and proteolytic activities of ClpE–wt and MD mutants (summarized in Table S2). ClpE–E373A and ClpE–F375S exhibited comparable or slightly reduced basal ATPase activities (Fig. 5*A*). FITC–casein and *Sa* ClpP–enhanced ATPase activities were similar (F375S) or higher (E373A) as compared with ClpE–wt. Proteolytic activities of both MD mutants were largely similar to ClpE–wt, though ClpE–E373A–*Sa* ClpP degraded FITC–casein twofold faster than ClpE–wt, whereas MDH disaggregation was approximately 1.7-fold and 2.6-fold slower for ClpE–E373A and ClpE–F375S, respectively (Fig. 5*B*). *B. subtilis* CtsR degradation remained unaffected by MD mutations (Fig. 5*B*). We infer that single-ring ClpE MD mutants are functional in all degradation assays, indicating that high ClpE activity is not tailored to a tetrahedron state.

### Substrate-specific targeting functions of the ClpE NTD

**Figure 6:**
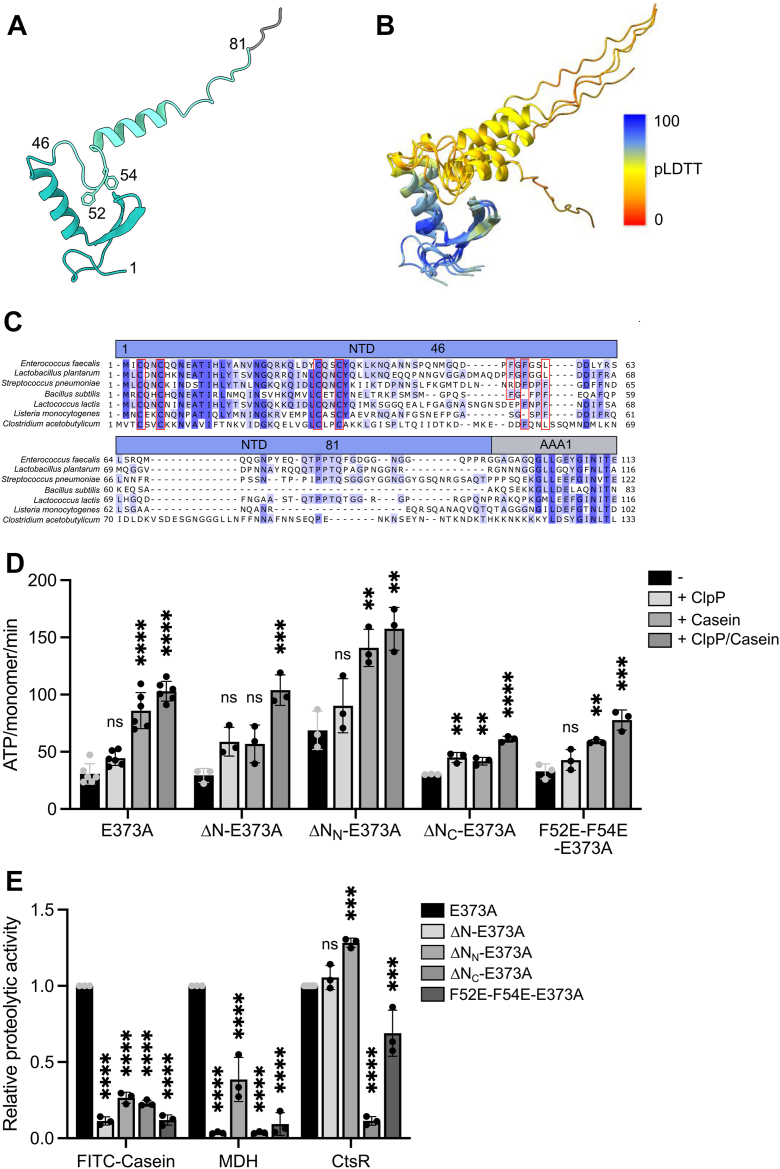
**Role of the N-terminal domain in ClpE activity.***A*, AlphaFold2 model of the ClpE N-terminal domain (N). The border between the N_N_ and N_C_ subdomains (residue 46) is indicated. Phe52 and Phe54 residues are shown. *B*, superposition of the top five AlphaFold2 predictions of the ClpE N-domain. The pLDDT plot to assess the AlphaFold2 prediction confidence is shown. *C*, sequence alignment of N-domains of indicated ClpE proteins. Conserved cysteine residues implicated in Zn^2+^ binding and phenylalanine residues are boxed in *red*. *D*, absolute ATPase activities of ClpE–E373A and its indicated N-domain mutant derivatives were determined in the absence and presence of *Sa* ClpP and casein. *E*, relative proteolytic activities of ClpE–E373A and its indicated N-domain mutant derivatives were determined for the substrates FITC–casein, aggregated MDH, and CtsR in the presence of *Sa* ClpP. Degradation rates were determined based on changes (%/min) in FITC–casein fluorescence and MDH turbidity and by SDS-PAGE analysis (CtsR). The proteolytic activity of ClpE E373A was set at 1 for each substrate, and relative proteolytic activities of ClpE mutants are shown. Error bars show standard deviations (n ≥ 3). Statistical analysis was performed by one-way ANOVA with Dunnett’s multiple comparison test (*D* and *E*). ns, not significant, ∗∗*p* < 0.01, ∗∗∗*p* < 0.001, and ∗∗∗∗*p* < 0.0001. MDH, malate dehydrogenase; pLDDT, predicted local distance difference test; *Sa*, Staphylococcus aureus.

ClpE does not require adaptor proteins to process PQC model substrates, raising the question of how ClpE targets the diverse substrates. ClpE harbors an NTD that is composed of a conserved N-terminal region (residues 1–46) and a more variable C-terminal part (residues 47–81) (Fig. 6, *A–C*). The N-terminal part exhibits sequence homology to the first NTD (N1) of the disaggregase ClpG and shows high structural similarity to the ClpG N1 core domain, consisting of an α-helix, an antiparallel β-sheet, and a Zn^2+^ binding center (Figs. 6*C* and S7*A*). The nonconserved C-terminal region of the ClpE NTD is largely disordered, also explaining the lower predicted local distance difference test values of the structural models (Fig. 6*B*). To explore the role of the NTD for ClpE activity, we deleted the complete NTD (Δ1–81, referred to as ΔN); however, we were not able to purify ΔN–ClpE from *Escherichia coli* cells after overproduction. We speculated that this variant might form large complexes *in vivo* that restrict accessibility of the C-terminal His_6_-tag used for purification. We probed whether formation of such complexes relies on the coiled-coil MD and generated ΔN–ClpE-E373A, which indeed could be purified with yields comparable to ClpE–E373A. We therefore dissected the function of the NTD in substrate targeting in the ClpE–E373A mutant background, as the MD mutant is functional in all proteolytic assays (Fig. 5*B*). We removed the entire NTD (ΔN–ClpE–E373A) and additionally deleted the conserved N-terminal (Δ1–46, referred to as ΔN_N_–E373A) and variable C-terminal (Δ47–81, referred to as ΔN_C_–E373A) parts. All NTD–E373A mutants formed complexes with radii similar to ClpE–E373A, and all associated with *Sa* ClpP as revealed by DLS measurements (Fig. S7, *B* and *C*). Accordingly, EM analysis showed that ΔN–ClpE–E373A formed single hexameric rings (Fig. S7*D*), excluding structural defects upon NTD deletion.

All NTD deletion constructs showed basal ATPase activities that were similar (ΔN–E373A, ΔN_C_–E373A) or increased (ΔN_N_–E373A) as compared with the ClpE–E373A reference (Fig. 6*D*). All mutants also displayed enhanced ATPase activity in the presence of *Sa* ClpP and casein, though the increase was lower for ΔN_C_–ClpE–E373A (Table S2).

FITC–casein degradation activities were reduced for all NTD mutants (Fig. 6*E*) and correlated with weakened binding of ClpE–E373A NTD deletion mutants to the substrate as observed in anisotropy experiments (Fig. S7*E*). We did not reach signal saturation in these experiments and thus were not able to calculate *K*_*D*_ values, yet the increase in FITC–casein anisotropy in the presence of NTD deletion mutants was strongly reduced as compared with the ClpE–E373A reference. We infer that the ClpE NTD is required for efficient FITC–casein binding and degradation. Residual binding and degradation of FITC–casein in the absence of the NTD suggests direct recognition and processing at the central ClpE pore site.

We next explored the contribution of the NTD to the degradation of aggregated MDH and *B. subtilis* CtsR (Fig. 6*E*). Unexpectedly, ΔN_N_–ClpE–E373A–*Sa* ClpP still processed MDH aggregates with 2.6-fold reduced efficiency as compared with ClpE–E373A–*Sa* ClpP. Thus, the conserved N_N_-subdomain is not essential for aggregate recognition by ClpE, in contrast to results obtained for the N1-domain of ClpG (26). ΔN–ClpE–E373A and ΔN_C_–ClpE–E373A were deficient in MDH disaggregation, suggesting a crucial role of the N_C_-subdomain in aggregate targeting (Fig. 6*E*). We obtained highly similar results when using *Ef* ClpP as a partnering peptidase (Fig. S7, *F–G*) or when monitoring refolding of aggregated MDH by the ClpE NTD mutants in the absence of ClpP (Fig. S7*H*). These findings confirm the autonomous activity of ClpE in substrate targeting and processing and peptidase interchangeability.

CtsR degradation was not affected by full NTD deletion, demonstrating substrate-specific degradation defects of ΔN–ClpE–E373A–*Sa* ClpP and indicating recognition of CtsR at the central pore site (Fig. 6*E*). Surprisingly, CtsR was not degraded by ΔN_C_–ClpE–E373A, indicating a dominant negative impact of the partial NTD deletion on ClpE activity. We speculate that the deletion of the disordered N_C_-subdomain, which links the N_N_-subdomain to the AAA1 ring, strongly reduces N_N_-mobility. This reduced mobility might block access of CtsR to the processing pore site.

Since the C-terminal part of the ClpE NTD is crucial for MDH disaggregation, we looked for conserved residues that might be implicated in aggregate recognition. We noticed a short stretch located right next to the conserved N-terminal core domain (N_N_) that harbors leucine and phenylalanine residues (Fig. 6, *A* and *C*) and generated ClpE–F52E–F54E–E373A to probe for a potential functional relevance. ClpE–F52E–F54E–E373A formed complexes with *Sa* ClpP (Fig. S7, *B* and *C*), excluding structural defects. Accordingly, its ATPase activities in the absence and presence of *Sa* ClpP were similar to ClpE–E373A (Fig. 6*D*). However, ATPase stimulation by casein was reduced (1.8- *versus* 2.8-fold, mutant *versus* E373A) and, similarly, FITC–casein degradation kinetics were severely affected by the F52E–F54E mutations (Fig. 6*E*). Importantly, ClpE–F52E–F54E–E373A hardly degraded aggregated MDH, whereas it retained substantial proteolytic activity toward *B. subtilis* CtsR (Fig. 6*E*). These findings indicate substrate-specific contributions of the Phe52–Phe54 residues and imply a crucial role in aggregate recognition.

### Zn^2+^ binding stabilizes the ClpE NTD

**Figure 7:**
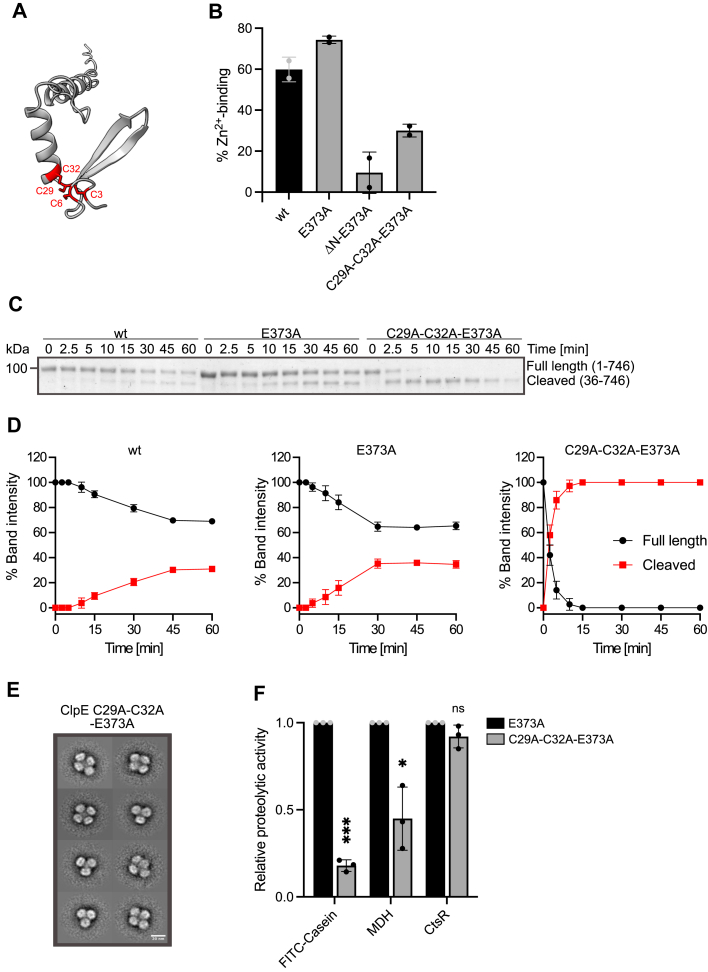
**Role of Zn^2+^ binding for N-domain integrity and ClpE activity.***A*, AlphaFold2 model of ClpE N-domain. Conserved cysteine residues implicated in Zn^2+^ binding are highlighted in *red*. *B*, Zn^2+^ binding to ClpE wt, ClpE–E373A, and its indicated N-domain mutant derivatives were determined by ICP–OES in two independent experiments. *C*, autoprocessing of ClpE wt, E373A, and C29A–C32A–E373A was monitored by SDS-PAGE and Coomassie staining. The positions of full-length and cleaved ClpE are indicated. *D*, band intensities of full-length and cleaved ClpE were quantified, and the cleavage kinetics and efficiencies were calculated (n = 3). *E*, 2D class averages of ClpE–C29A–C32A–E373A based on negative-stain EM. The scale bar represents 20 nm. *F*, relative proteolytic activities of ClpE–E373A and its indicated N-domain mutant Cys29–Cys32 were determined for the substrates FITC–casein, aggregated MDH, and CtsR in the presence of *Sa* ClpP. Degradation rates were determined based on changes (%/min) in FITC–casein fluorescence and by Western blot (MDH) and SDS-PAGE analysis (CtsR). The proteolytic activity of ClpE–E373A was set to 1 for each substrate, and the relative proteolytic activities of ClpE–C29A–C32A–E373A are shown. Error bars show standard deviations (*B*: n = 2; *D* and *F*: n = 3). Statistical analysis was performed by an unpaired *t* test with Welch’s correction (*F*). ns, not significant, ∗*p* < 0.05, and ∗∗∗*p* < 0.001. ICP–OES, inductively coupled plasma optical emission spectrometry; MDH, malate dehydrogenase; *Sa*, Staphylococcus aureus.

The ClpE NTD includes a putative Zn^2+^ binding center composed of four conserved cysteine residues (C3, C6, C29, and C32) (Fig. 7*A*). We mutated C29 and C32 residues to alanines in the ClpE–E373A MD mutant background and determined Zn^2+^ binding by inductively coupled plasma optical emission spectrometry (ICP–OES) measurements. ClpE–wt and ClpE–E373A bound Zn^2+^; though not all subunits of a hexamer were loaded, as Zn^2+^ occupancy was 60% and 74%, respectively (Fig. 7*B*). Zn^2+^ binding was largely abrogated upon full NTD deletion (ΔN–ClpE–E373A) and reduced in the case of ClpE–C29A–C32A–E373A (Zn^2+^ occupancy of 30% as compared with 74% for ClpE–E373A) (Fig. 7*B*).

When probing for proteolytic activity of ClpE–C29A–C32A–E373A–Sa ClpP, we noticed fast and quantitative autoprocessing of ClpE–C29A–C32A–E373A, leading to the accumulation of a truncated ClpE protein (Fig. 7, *C* and *D*). Fast autoprocessing of ClpE–C29A–C32A–E373A was also observed in the presence of substrate (Fig. S8, *A* and *B*). We also observed autoprocessing for ClpE–wt and ClpE–E373A, yet here the truncated fragments were generated more slowly and less efficiently (Figs. 7, *C* and *D* and S8, *A* and *B*). Partial autoprocessing of ClpE–wt and ClpE–E373A is consistent with incomplete Zn^2+^ loading determined by ICP–OES (Fig. 7*B*). The formation of a truncated ClpE product was also observed *in vivo* not only for the *L. lactis* ClpE–C29S mutant but also for ClpE–wt upon oxidative stress conditions (37, 49).

We characterized the nature of the truncated ClpE fragment by mass spectrometry (MS) and determined that it is lacking the N-terminal residues 1 to 35 (Fig. S8*C*). This finding suggests that loss of Zn^2+^ binding in ClpE–C29A–C32A–E373A causes unfolding of the N_N_-subdomain and its subsequent autoprocessing. Notably, autoprocessing is not processive but arrests after threading of almost the entire NTD and before the start of the AAA1 domain. This calculation takes the degraded (residues 1–35) and the threaded polypeptide segment (∼residues 36–76) located between the entrance of the ClpE threading channel and the proteolytic active sites of ClpP (distance ∼140 Å, corresponding to approximately 35–40 residues) into account (Fig. S8*D*). We suggest that threading arrests once unfolding of the AAA1 domain starts, as this will affect ClpE ring integrity, impairing ATPase and threading activities. Does threading of the unfolded N_N_-subdomain occur in *cis* by the same ClpE hexamer or in *trans* by a second ClpE hexamer? DLS measurements revealed the formation of larger complexes of ClpE–C29A–C32A–E373A as compared with ClpE–E373A (radius of 20 nm *versus* 10 nm, respectively) (Fig. S8, *E* and *F*). Addition of *Sa* ClpP led to formation of very large, heterogenous complexes (Fig. S8, *E* and *F*). These features of ClpE–C29A–C32A–E373A oligomerization are reminiscent of ClpE–wt. Accordingly, EM analysis revealed that ClpE–C29A–C32A–E373A hexamers interact to form larger assemblies (Figs. 7*E* and S8*G*). Those assemblies included tetrahedrons, but they were more heterogenous as compared with ClpE–wt. These findings point to a recognition of an unfolded N_N_-subdomain by another ClpE ring *in trans* causing the formation of hexamer-based assemblies since MD–MD interactions can be excluded because of the E373A mutation. We next probed for proteolytic activities of ClpE–C29A–C32A–E373A–*Sa* ClpP, and its activity profile was similar to ΔN_N–_ClpE–E373A, consistent with cleavage of the N_N_-subdomain (Fig. 7*F*).

### Interplay of NTD and MD controls ClpE activity and assembly *in vivo*

**Figure 8:**
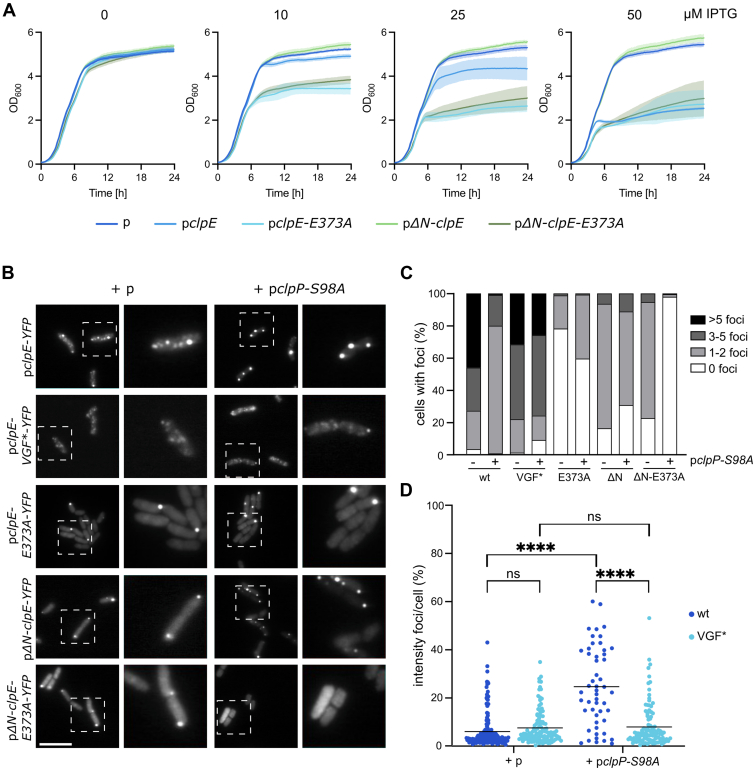
**Proteolytic activities and localizations of ClpE–*Sa* ClpP complexes in *Escherichia coli* cells.***A*, *E. coli* cells expressing *Sa clpP* and harboring indicated plasmid-encoded *clpE* alleles under control of an IPTG-regulatable promoter were grown in the presence of 0 to 50 μM IPTG at 30 °C for 24 h. Standard deviations are shown as shaded areas. n = 3. *p*: empty vector control. *B*, cellular localization of ClpE–YFP fusions in *E. coli* cells expressing indicated plasmid-encoded *clpE-yfp* alleles under control of an IPTG-regulatable promoter were grown at 30 °C in the presence of 25 μM (*clpE*, *clpE–VGF∗*, and *clpE–E373A*) or 100 μM *(ΔN–clpE*, *ΔN–clpE–E373A*) IPTG. Cells additionally harbored *pclpP–S98A* or a respective empty vector control (*p*). ClpE–YFP localizations were determined in the absence and presence of *Sa* ClpP–S98A by fluorescence microscopy. *Boxed cells* are additionally shown as an enlarged image. The scale bar represents 5 μm. *C*, percentage of cells harboring the indicated number of ClpE–YFP foci was determined in the absence and presence of *Sa* ClpP–S98A (n > 80, two independent biological replicates). *D*, relative intensities of ClpE–YFP foci (% of total cellular fluorescence) were determined in the absence (*p*) and presence of *Sa* ClpP–S98A (n > 25, two independent biological replicates). Statistical analysis was performed by an unpaired *t* test with Welch’s correction. ns, not significant, ∗∗∗∗*p* < 0.0001. *Sa*, Staphylococcus aureus.

We probed for proteolytic activity of ClpE constructs upon production in *E. coli ΔclpB* cells. Here, we reasoned that overproduction of an active ClpE–*Sa* ClpP protease will create toxicity as observed before for constitutively active *Sa* ClpC mutants and *M. tuberculosis* ClpC1 (22, 24). We expressed ClpE, ΔN–ClpE, and their MD mutant derivatives in *E. coli* cells from IPTG-controlled promoters either alone or together with *Sa* ClpP. Coexpression of *Sa* ClpP created toxicity for all ClpE constructs, except ΔN–ClpE on LB plates (Fig. S9*A*). Toxicity increased at higher incubation temperatures (40 °C) and at higher IPTG concentrations. *E. coli ΔclpB* does not exhibit a temperature-sensitive growth phenotype; we therefore consider it unlikely that increased toxicity at 40 °C is indirectly caused by the absence of ClpB and an increased stress load. Toxicity was not observed upon sole expression of either *Sa* ClpP or the ClpE constructs, demonstrating that the overproduction of an active protease is lethal to *E. coli* cells (Figs. 8*A* and S9*A*). Accordingly, we did not observe cooperation between ClpE and *E. coli* ClpP *in vitro* (Fig. S9*B*), rationalizing why coexpressing of cooperating *Sa* ClpP is necessary for *in vivo* toxicity. Production levels of ClpE–wt and ClpE–MD mutants in the absence of *Sa* ClpP were similar, whereas all ΔN–ClpE constructs were produced at lower levels (Fig. S9*C*). Coexpression of *Sa* ClpP strongly reduced expression levels of ClpE constructs that create toxicity, while not affecting nontoxic ΔN–ClpE (Fig. S9*C*). Differences in toxicities caused by expression of ΔN–ClpE and its MD mutant derivatives are thus not caused by increased expression of the toxic variants. These findings confirm that ClpE functions autonomously and does not require partner proteins for being active *in vivo*. Furthermore, they suggest a role of the ClpE NTD in controlling ClpE activity *in vivo*, as its deletion renders ClpE nontoxic. This role of the NTD must involve the coiled-coil MD, since mutating MD residues implicated in MD–MD interactions restore toxicity (Fig. S9*A*).

Toxicities of ClpE–wt and its MD mutant derivatives were comparable on LB plates (Fig. S9*A*). We additionally expressed the constructs together with *Sa* ClpP in liquid LB medium at 30 °C in the presence of low (0–50 μM) IPTG concentrations and recorded their growth curves (Fig. 8*A*). Here, expression of ClpE–E373A and ΔN–ClpE–E373A impaired growth at lower IPTG concentrations (10–25 μM), whereas ClpE–wt had a similar inhibitory effect only at 50 μM IPTG. Levels of ClpE–E373A and *Sa* ClpP were strongly reduced as compared with ClpE–wt–*Sa* ClpP, in agreement with an increased toxicity of ClpE–E373A–*Sa* ClpP complexes (Fig. S9*D*). Since the proteolytic activities of ClpE–wt and ClpE–E373A are similar *in vitro*, we speculated that differences in their cellular localizations might contribute to their differing toxicities.

We explored the cellular localizations by creating C-terminal fusions to monomeric YFP. The fusion constructs retained toxicity in *E. coli ΔclpB* cells upon coexpression with *Sa* ClpP (Fig. S9, *E* and *F*), demonstrating functionality. We only noticed a slightly reduced toxicity for ΔN–ClpE–E373A–YFP. ClpE–YFP formed multiple foci upon moderate expression in the absence of *Sa* ClpP (Figs. 8, *B* and *C* and S10*A*). These foci represent MD-dependent assemblies but no protein aggregates, as foci formation was strongly reduced for ClpE–E373A–YFP (Fig. 8, *B* and *C*). We next determined the impact of *Sa* ClpP on ClpE–YFP localization by coexpressing the proteolytically inactive *Sa* ClpP–S98A variant to exclude that ClpE–YFP localization is affected by an induced toxicity of an active ClpE–YFP–ClpP protease. ClpE–YFP formed fewer but more intense foci in the presence of *Sa* ClpP–S98A (24.8% mean foci intensity compared with 6.1% without ClpP–S98A) indicating the formation of larger assemblies (Fig. 8, *B*–*D*). In contrast, the presence of *Sa* ClpP–S98A had little effect on the localization of ClpE–E373A–YFP. This suggests that the formation of very intense ClpE–YFP–*Sa* ClpP foci involves MD interactions (Fig. 8, *B* and *C*). This *in vivo* finding is reminiscent of the large meshwork formed by ClpE tetrahedrons and *Sa* ClpP *in vitro* (Fig. 4, *B* and *C*). To demonstrate that complex formation with *Sa* ClpP–S98A triggers ClpE–YFP relocalization, we monitored the localization of ClpE–VGF∗–YFP, which is deficient in ClpP interaction by harboring a mutated P-loop (602VGF604 mutated to 602GGR604). Coexpression of ClpE–VGF∗–YFP with *Sa* ClpP did not create toxicity in *E. coli* cells, confirming its deficiency in ClpP interaction (Fig. S10, *B* and *C*). ClpE–VGF∗–YFP formed multiple foci per cell, like ClpE–YFP; however, its localization pattern was unaffected upon *Sa* ClpP–S98A coexpression (Fig. 8, *B* and *C*). Accordingly, ClpE–VGF∗–YFP foci intensities were similar in the absence and presence of *Sa* ClpP–S98A (7.6 and 8.0% mean foci intensities, respectively) (Fig. 8*D*). This demonstrates that very large ClpE–YFP assemblies are triggered by and include ClpP–S98A (Fig. 8, *B*–*D*). To exclude that such assemblies only form by proteolytic inactive ClpE–YFP–*Sa* ClpP–S98A complexes, we also analyzed ClpE–YFP localization upon coexpression with functional *Sa* ClpP. We again observed the formation of less but more intense ClpE–YFP foci, whereas the pattern of ClpE–VGF∗–YFP remained unaltered (Fig. S10, *D–F*).

We finally monitored the localization of ΔN–ClpE–YFP and its MD mutant derivative. We used higher IPTG concentrations for these constructs (100 μM instead of 25 μM) to adjust comparable expression levels. ΔN–ClpE–YFP formed fewer foci as compared with ClpE–YFP, and foci formation was not affected by *Sa* ClpP–S98A presence (Fig. 8, *B* and *C*). This suggests an altered assembly state of ΔN–ClpE-YFP, which is not able to form complexes with *Sa* ClpP, explaining why their coexpression does not create toxicity. ΔN–ClpE–E373A–YFP formed foci in the absence of *Sa* ClpP, in contrast to ClpE–E373A–YFP, again pointing to a role of the NTD in modulating ClpE assembly. Notably, coexpression of *Sa* ClpP led to disappearance of ΔN–ClpE–E373A–YFP foci, and only a diffuse fluorescence signal was observed (Fig. 8, *B* and *C*). The impact of *Sa* ClpP on ΔN–ClpE–E373A–YFP localization indicates complex formation, and, accordingly, their coexpression creates toxicity (Figs. 8*A* and S9*D*). These findings indicate that ΔN–ClpE–YFP foci do not represent protein aggregates but an alternative assembly state involving MD interactions.

## Discussion

In the present work, we provide the first biochemical characterization of the bacterial ClpE–ClpP AAA+ protease. We show that ClpE–ClpP functions as an autonomous protease, which does not require assistance of adaptor proteins for activation or targeting to PQC substrates, unlike ClpC–ClpP. The standalone activity of ClpE is consistent with the monocistronic organization of *clpE* genes in bacterial genomes. In contrast, the *clpC* gene is frequently co-organized with *mcsA* and *mcsB* genes encoding for a co-operating adaptor (11, 21). Our findings do not exclude the existence of adaptors that target ClpE to specific, non-PQC substrates. Such a role has been recently suggested for RfaA, a largely uncharacterized protein, which can target ClpE–ClpP to AccC, a subunit of the acetyl-CoA carboxylase complex (50).

We show that ClpE–ClpP processes misfolded and aggregated proteins with high efficiencies, similar to adaptor-activated ClpC–ClpP or the potent disaggregase ClpG. These findings can explain the phenotypes of *clpE* mutant cells, which show an accumulation of aggregated proteins and defects in shutting off cellular stress responses, reflecting delayed damage removal (12, 37). Our *in vitro* analysis supports a central role of the ClpE–ClpP protease in bacterial stress adaptation by clearance of misfolded and aggregated proteins. ClpE–ClpP will complement the PQC activities of the AAA+ protease ClpC–ClpP and the ClpB/DnaK or ClpL disaggregases as it coexists with these PQC factors in diverse bacilli (Fig. S11). How protein disaggregation is orchestrated in bacteria encoding for refolding (ClpB/DnaK, ClpL) and degrading (ClpE–ClpP) disaggregases remains to be elucidated. The high autonomous proteolytic activity of ClpE–ClpP might explain why in *B. subtilis* ClpE accumulation is restricted to the immediate phase after stress application (35). Accordingly, we show that expression of ClpE–*Sa* ClpP at increased levels in *E. coli* cells is toxic.

ClpE does not form a resting state like ClpC but hexameric rings, which interact *via* their coiled-coil MDs to form tetrahedrons. Such complexes have also been described for ClpC upon activation by either the antibacterial peptide cyclomarin A (23) or by substrates harboring phosphorylated arginines as specific degrons (46). Furthermore, *L. monocytogenes* ClpL rings can form tetrahedrons, however with limited efficiency and in a species-specific manner (47, 48). Thus, ClpE differs from ClpC and ClpL by pronounced and factor-independent tetrahedron formation. We did not observe strong differences in proteolytic activities between ClpE–wt and MD mutants that only form single rings, though an approximately twofold reduction in disaggregation activity was determined for MD mutants. We infer that both assembly states are functional. It remains to be determined whether substrate presence triggers a transition from a tetrahedron to a hexamer or *vice versa*.

Why is ClpE forming tetrahedral assemblies? Tetrahedron formation might play a role in spatially controlling ClpE–ClpP activity *in vivo*. We show that ClpE–ClpP complexes form a large meshwork *in vitro*, resulting from the presence of four ClpP docking sites in a ClpE tetrahedron and two ClpE docking sites in ClpP. Similarly, the size of MD-dependent ClpE–YFP foci was strongly increased upon coexpression of *Sa* ClpP in *E. coli* cells. We consider the presence of potential ClpE adaptor proteins in *E. coli* unlikely; thus, the heterologous *E. coli* system reports on the intrinsic capability of ClpE–*Sa* ClpP complexes to form large meshworks *in vivo*. It is possible that ClpE–ClpP meshwork formation and cellular localization are regulated by adaptors in *Ef*, and modulating the ClpE–ClpP assembly state could represent a novel mode of proteolysis control. Notably, large, polar clusters formed by fluorescently tagged ClpE–ClpP proteases were also observed in heat-shocked *B. subtilis* cells (6), supporting the usefulness of the *E. coli* system. We speculate that such clusters allow for localized and confined proteolysis in bacterial cells, increasing target specificity while protecting, for example, nascent polypeptides from unwanted proteolysis by spatially separating protein synthesis from degradation. Indeed, expression of the ClpE–Y344A MD mutant (equivalent to *Ef* ClpE–F375S) in *B. subtilis* is highly toxic, in contrast to ClpE–wt (22). We also noticed an increased toxicity of ClpE MD mutants as compared with ClpE–wt when expressed together with *Sa* ClpP in *E. coli* cells. This enhanced toxicity correlates with a diffuse localization of respective protease complexes, supporting a role of large ClpE–ClpP clusters in restricting protease activity *in vivo*.

How does ClpE gain target specificity? We show that the NTD has substrate-specific functions and is required for efficient degradation of soluble misfolded and aggregated model substrates. In contrast, degradation of the *B. subtilis* CtsR repressor is NTD independent, implying its direct recognition by the central pore site. We show that the N_N_-subdomain is crucial for efficient binding and processing of the model substrate FITC–casein. Surprisingly, deletion of the N_N_-subdomain only decreased disaggregation activity but did not abolish it and is thus not essential for ClpE disaggregation activity. This is in contrast to the role of the homologous N1-domain in the case of ClpG-mediated disaggregation (26). This finding is unexpected as both domains share sequence and structural homology. We speculate that the contribution of the N_N_-subdomain to aggregate recognition might be more relevant *in vivo* when ClpE has to compete with other PQC factors for aggregate binding. Thus, it will be relevant to determine the disaggregation activity of ClpE NTD mutants in an *in vivo* setting. The N_C_-subdomain is crucial for the degradation of all model substrates tested. This implies a central role in substrate binding, though the deficits of ΔN_C_–E373A–ClpE in degrading CtsR point to additional, indirect effects impacting its activity. We identified a short stretch enriched in phenylalanine residues (Phe52–Phe54) located at the beginning of the N_C_-subdomain and demonstrated crucial and substrate-specific contributions to ClpE activity. The hydrophobic character of this short motif implies a recognition principle of misfolded and aggregated proteins *via* hydrophobic interactions.

We were not able to purify and characterize ΔN–ClpE for unknown reasons. ΔN–ClpE did not create toxicity when coexpressed with *Sa* ClpP in *E. coli* cells, and the localization pattern of ΔN–ClpE–YFP did not change upon coexpression of *Sa* ClpP–S98A. This implies an altered ΔN–ClpE assembly state that is incompatible with ClpP interaction. Toxicity of ΔN–ClpE was restored upon additional mutation of the MD (E373A), suggesting that such altered assembly state involves MD interactions and is not simply caused by protein aggregation. These findings imply an interplay between ClpE NTDs and MDs in controlling the activity and assembly state of ClpE *in vivo*.

## Experimental procedures

### Strains and plasmids

All strains and plasmids used in this study are summarized in Table S1. ClpE deletion mutants were generated by PCR, and point mutants were constructed by QuikChange one-step site-directed mutagenesis. All mutations were verified by sequencing. *E. coli* cells were grown in LB medium at 30 °C containing appropriate antibiotics with an agitating speed of 120 rpm. For protein overproduction, 2x YT medium (16 g/l tryptone, 10 g/l yeast extract, 5 g/l NaCl, adjusted to pH 7) was used. *E. coli* XL1 blue was used for cloning and retaining of plasmids requiring kanamycin at 50 μg/ml and ampicillin at 100 μg/ml for plasmid propagation.

### Proteins

*Ef* ClpE was purified after overproduction from *E. coli ΔclpB::kan* cells using either pUHE21-derived expression vectors (wt, E373A, F375S, Δ2–46–E373A, Δ47–81–E373A, and C29A–C32A–E373A) or pDS56-derived expression vectors (ΔN–E373A). *Ef* ClpP and *Sa* McsA and McsB and *P. aeruginosa* ClpG were purified after overproduction in *E. coli* BL21 cells using pET24a-derived expression vectors. *Sa* ClpP, ClpC, and MecA were purified after overproduction from *E. coli ΔclpB::kan* cells using pDS56-derived expression vectors (22). Intact protein mass determination documented purity of *Sa* ClpP and *Ef* ClpP, excluding copurification of endogenous *E. coli* ClpP (Fig. S12).

ClpE, ClpC, MecA, McsA, ClpP, and ClpG proteins harbor a C-terminal His6-tag and were purified using Ni–IDA (Macherey–Nagel) following the instructions provided by the manufacturer. In short, cell pellets were resuspended in buffer A (50 mM NaH_2_PO_4_, 300 mM NaCl, 5 mM β-mercaptoethanol, pH 8.0) supplemented with protease inhibitors (Roche). After cell lysis using a French press, the cell debris was removed by centrifugation at 17,000*g* for 60 min at 4 °C, and the cleared lysate was incubated with 0.6 to 0.8*g* Protino Ni–IDA resin for 20 min at 4 °C. Afterward, the resin was transferred into a plastic column and washed once with buffer A. His-tagged proteins were eluted by the addition of buffer A supplemented with 250 mM imidazole. Subsequently, pooled protein fractions were subjected to SEC (Superdex S200, Amersham or Superdex S75, Amersham) in buffer B (50 mM Hepes–HCl [pH 7.5], 150 mM KCl, 10 mM MgCl_2_, 10% [v/v] glycerol, and 2 mM DTT) for ClpE (wt and variants), in buffer B1 (50 mM Tris–HCl, 150 mM KCl, 20 mM MgCl_2,_ 5% [v/v] glycerol, and 2 mM DTT) for *Ef* ClpP and in buffer B2 (50 mM Tris–HCl, 50 mM KCl, 20 mM MgCl_2,_ 5% [v/v] glycerol, and 2 mM DTT) for *Sa* ClpP, ClpC, MecA, McsA, and *P. aeruginosa* ClpG.

McsB harboring a C-terminal His6-tag was purified using a 1 ml HisTrap column (GE Healthcare). Cells were resuspended in buffer (50 mM NaH_2_PO_4_, 500 mM NaCl, 20 mM imidazole, 5 mM β-mercaptoethanol, pH 8.0) supplemented with protease inhibitors (Roche) and lysed as described above. Proteins were eluted by applying a linear gradient to 500 mM imidazole over 50 column volumes (CVs). Fractions including pure McsB were pooled and dialyzed against high salt dialysis buffer (20 mM NaH_2_PO_4_, 500 mM NaCl, 10% [v/v] glycerol, 5 mM β-mercaptoethanol, pH 7.4). Purification of ΔN–ClpE failed as binding to Ni–IDA was poor, and the eluted protein was aggregation prone.

*B. subtilis* CtsR was purified after overproduction from *E. coli* AG1 cells using a pQE32-derived expression vector. Cells were resuspended at room temperature in B-PER buffer (ThermoFisher) supplemented with cOmplete EDTA-free protease inhibitors (5 ml of buffer per 1 g of cells). The resuspended pellet was then incubated by rotation for 30 min at room temperature. Cell debris was pelleted at 12,000 rpm for 10 min at 4 °C, and the supernatant was filtered (0.45 μm). The cell extract was then mixed with 1 volume of washing buffer (50 mM potassium phosphate buffer [pH 7.5], 500 mM NaCl, and 50 mM imidazole). The cell extract was then loaded on the HisTrap Ni–NTA FF column (CV 1 ml) pre-equilibrated with 20 CVs of washing buffer. The sample was loaded using the sample pump at 1 ml/min. The column was then washed with buffer A for 20 CVs. Step-wise elution of 10%, 20%, 30%, 40%, 50%, and 100% elution buffer (50 mM potassium phosphate buffer [pH 7.5], 500 mM NaCl, and 1 M imidazole) was performed. For each step, two CVs and 0.2 ml fractions were collected. The peak was observed at 40% to 50% of elution buffer. The fractions representing the peak were collected (circa 4 ml) and concentrated to a final volume of 0.5 ml using a Vivaspin 500 10 K filter at 4 °C. The concentrated sample was then loaded on a Superdex 75 Increase 10/300 GL pre-equilibrated with two CVs of elution buffer. Fractions (0.25 ml) were collected.

Pyruvate kinase (PK) of rabbit muscle, FITC–casein from bovine milk, and MDH of pig heart muscle were purchased from Sigma. Protein concentrations were determined with the Bradford assay (Bio-Rad).

### Biochemical assays

Buffer X (50 mM Tris–HCl [pH 6.5], 50 mM KCl, 20 mM MgCl_2_, and 2 mM DTT) was used for all biochemical assays if not mentioned otherwise.

### ATPase assay

Rates of ATP hydrolysis were determined by a coupled-colorimetric assay as described before (51). Oxidation of NADH to NAD^+^ was monitored by measuring the absorbance at a wavelength of 340 nm.

ATPase activity measurements were performed at final concentrations of 0.5 μM ClpE, 1 μM *Sa*/*Ef* ClpP, 5 μM casein, and an ATP-regenerating system (PK–lactate dehydrogenase mix: 250 μM NADH, 500 μM phosphoenolpyruvate [PEP], 1/20 [v/v] PK/lactate dehydrogenase [Sigma–Aldrich]) in buffer X supplemented with 2 mM ATP at 30 °C in a transparent 96-well plate (Greiner) using a CLARIOstar^plus^ plate reader (BMG Labtech).

The ATP hydrolysis rate for at least three biological replicates was calculated according to the following equation:

$$\text{ATPase}\;\text{rate}=\frac{1}{\varepsilon_{\text{NADH}}\times C_{\text{ClpE}}\times d}\times \frac{\Delta A_{340\;\text{nm}}}{\Delta t}$$

ε_NADH_—molar absorption coefficient of NADH at a wavelength of 340 nm (6220 M^−1^cm^−1^).

C_ClpE_—final concentration of ClpE (0.5 μM).

d—optical path length (1 cm).

ΔA_340 nm_/Δ*t*—slope of the linear decline in absorption at a wavelength of 340 nm.

### Degradation assays

Proteolytic activity was determined using fluorescein-labeled casein (FITC–casein). The increase of fluorescence upon FITC–casein degradation was monitored at 483-14 nm and 530-30 nm as excitation and emission wavelengths, respectively, in black 96-well plates (Greiner) using a CLARIOstar^plus^ plate reader.

All degradation measurements were performed in buffer X at 30 °C at final concentrations of 0.5 to 1 μM ClpE or ClpC, 0.75 μM MecA, 0.75 to 1.5 μM McsA–McsB, and 1 to 2 μM *Sa*/*Ef* ClpP. Substrates were used at the following concentrations: 0.15 μM FITC–casein, 1 μM aggregated MDH, and 5 μM CtsR. MDH aggregates were generated by incubation of native MDH for 30 min at 47 °C. Degradation reactions included an ATP regenerating system (2 mM ATP, 3 mM PEP, and 20 ng/μl PK [Sigma–Aldrich]).

FITC–casein degradation was analyzed by setting the initial raw fluorescence signal to 100%. The FITC–casein fluorescence gain (% min^−1^) was calculated by determining the initial slopes of the linear fluorescence signal increase. Degradation of aggregated MDH was monitored by Western blot analysis using MDH-specific antibodies. MDH band intensities were quantified by ImageJ2 (https://imagej.net/software/imagej2/), and the slopes of the linear decrease in MDH band intensities were determined. Degradation of *B. subtilis* CtsR was monitored by Coomassie-stained SDS-gels. CtsR band intensities were quantified by ImageJ2, and the slopes of the linear decrease in CtsR band intensities were determined.

LY-AMC (200 μM; succinyl-L-leucyl-L-tyrosyl-AMC) was incubated with 1 μM *Sa*/*Ef* ClpP in buffer X in the absence or presence of 1.5 μg/ml ADEP-1. LY-AMC proteolysis was monitored by determining LY-AMC fluorescence intensity using 355 nm and 460 nm as excitation and emission wavelengths, respectively, using an LS55 spectrofluorometer (PerkinElmer).

### MDH disaggregation

MDH disaggregation was monitored by turbidity measurements using an LS55 spectrofluorometer (PerkinElmer) at 600 nm excitation and emission wavelengths. MDH (2 μM) was heat-aggregated at 47 °C for 30 min in buffer X (50 mM Tris [pH 6.5], 50 mM KCl, 20 mM MgCl_2_, and 2 mM DTT). Aggregated MDH was mixed 1:1 with disaggregases (final concentrations: 1 μM ClpE or 1 μM ClpG), and disaggregation reactions were started by the addition of an ATP regenerating system (2 mM ATP, 3 mM PEP, and 20 ng/μl PK) and performed at 30 °C in buffer X.

MDH disaggregation was additionally followed by monitoring MDH refolding. These experiments additionally included 1 μM GroEL–GroES to facilitate refolding of disaggregated MDH. MDH activity was determined at indicated time points by measuring delta absorbance at 340 nm/min in a photometer. Here, 10 μl of disaggregation reaction was mixed with 690 μl of MDH assay buffer (150 mM potassium phosphate, 0.5 mM oxaloacetate, 0.28 mM NADH, 2 mM DTT, pH 7.6), and absorbance at 340 nm was recorded for 30 s.

### Anisotropy measurements

FITC–casein binding was monitored by anisotropy measurements. FITC–casein (100 nM) was preincubated with varying concentrations of ClpE (0.04–10 μM) in buffer X in the presence of 2 mM ATPγS at 25 °C for 10 min. Changes in fluorescence polarization were determined with a CLARIOstar^plus^ plate reader by measuring the polarization at 482-16 nm and 530-40 nm as excitation and emission wavelengths, respectively.

### GA crosslinking

For GA crosslinking, 1 μM ClpE (WT or mutants) or 1 μM ClpB was preincubated with 2 mM ATPγS at 25 °C for 10 min in buffer Y (50 mM Hepes [pH 7.5], 25 mM KCl, 10 mM MgCl_2_, and 2 mM DTT). Crosslinking was initiated by the addition of GA (Sigma) to a final concentration of 0.1%. Samples were taken after 0/2/10 min (time point 0 was taken prior to GA addition), and crosslinking was quenched by adding Tris (pH 7.5) to a final concentration of 50 mM. In addition, SDS sample buffer was added, and after boiling for 5 min at 95 °C, samples were subjected to SDS-PAGE analysis. Gels were stained with SYPRO Ruby Protein Gel Stain (Thermo Scientific).

### Dynamic light scattering

DLS was performed with a Prometheus Panta (NanoTemper) at 30 °C using Prometheus standard capillaries (NanoTemper). The hydrodynamic radius of ClpE complexes in the absence and presence of ClpP was determined using 3 μM ClpE (WT or mutants) and 4.5 μM *Sa*/*Ef* ClpP in buffer X. Samples were preincubated for 5 to 10 min at room temperature with 2 mM ATPγS. Three replicates were taken for each sample, and single capillaries were scanned 10 times. Hydrodynamic radii were determined from particle size distribution peak values using the Prometheus Panta Analysis software (version 1.4.3).

### Analytical SEC

Complex formation of ClpE WT and respective variants was monitored *via* analytical SEC. SEC was performed at 4 °C using an ÄKTA pure (Cytiva) equipped with a Superose 6 Increase 10/300 GL column (Cytiva), pre-equilibrated with buffer X supplemented with 2 mM ATP. ClpE (3 μM; WT or mutants) (in the presence and absence of 4.5 μM *Sa* ClpP) was preincubated with 2 mM ATPγS at room temperature for 5 min, and 200 μl of the sample was loaded onto the column. Aliquots were taken and subjected to SDS-PAGE analysis. Gels were stained using SYPRO Ruby Protein Gel Stain.

### Inductively coupled plasma optical emission spectrometry

Zn^2+^ binding of ClpE WT and mutants was determined *via* ICP–OES. About 0.9 ml of 7 μM ClpE WT and F375S, 4 μM E373A and ΔN–E373A, and 2 μM C29A–C32A–E373A (10 mM Tris–HCl [pH 7.5], 20 mM KCl, 5% glycerol, and 2 mM DTT) were incubated with 2 ml HNO_3_ (65%, for analysis; NeoFroxx GmbH) for 1 h at 90 °C. Afterward, double-distilled water was added to a final volume of 10 ml. Zn^2+^ presence was determined using Agilent 720 ICP–OES at a wavelength of 213,857 nm. For calibration, ICP multielement standard solution IV (Merck KGaA) was used.

### Mass spectrometry

Coomassie-stained bands were manually excised from the SDS-gel. The in-gel digestion was performed as described earlier (52, 53). Samples were suspended in 0.1% TFA and analyzed using an Ultimate 3000 liquid chromatography system coupled to an Orbitrap QE HF (Thermo Fisher) as described before (53). Briefly, peptides were separated in a 30 min linear gradient starting from 3% B and increasing to 23% B over 25 min and to 38% B over 5 min, followed by washout with 95% B. The mass spectrometer was operated in data-dependent acquisition mode, automatically switching between MS and MS2. MS spectra (*m/z* 400–1600) were acquired in the Orbitrap at 60,000 (*m/z* 400) resolution, and MS2 spectra were generated for up to 15 precursors with a normalized collision energy of 27 and an isolation width of 1.4 *m/z*. The MS/MS spectra were searched against the UniProt *E. coli* (UPUP000000625), *Ef* (UP000001415), a customized FASTA sequence of a protein of interest, and a customized contaminant database using Proteome Discoverer 2.5 (Thermo Fisher Scientific) with Sequest HT. The fragment ion mass tolerance was set to 0.02 Da and the parent ion mass tolerance to 5 ppm. Trypsin was specified as an enzyme. The following variable modifications were allowed: Oxidation (M), Deamidation (N, Q), Acetylation (N terminus), Met-loss (M), and a combination of Met-loss and acetylation (N terminus), whereas Carbamidomethylation (C) was set as a fixed modification. Peptide quantification was done using a precursor ion quantifier node with the top N average (n = 3) method set for protein abundance calculation.

The intact masses of proteins were determined by electrospray ionization MS using a quadrupole time-of-flight mass spectrometer (Maxis, Bruker Daltonics) following desalting *via* HPLC (Agilent Technologies). A total of 200 μl of protein sample at a concentration of 0.25 μM was loaded onto a reversed-phase trapping column (0.8 × 2 mm, Poros R1; Applied Biosystems). After washing for 3 min with 0.3% formic acid at a flow rate of 300 μl/min, proteins were eluted using a mobile phase consisting of 40% isopropanol, 5% acetonitrile, and 0.3% formic acid at a flow rate of 40 μl/min.

Mass spectra were acquired in positive ion mode over an *m/z* range of 500 to 4000 using full-scan acquisition. The spectral rate was set to 2 Hz, and rolling average smoothing was applied with a 2× setting. Mass spectra were deconvoluted using the MaxEnt algorithm implemented in Compass DataAnalysis 4.2 software (Bruker Daltonics).

### Negative-stain EM

Negative staining, data collection, and processing were performed as described previously (54). In brief, 5 μl sample were applied to a glow-discharged grid with a 6 to 8 nm thick layer of continuous carbon. After incubation for 5 to 60 s, the sample was blotted on a Whatman filter paper 50 (1450-070) and quickly washed with three drops of water. Samples on grids were stained with 2.5% aqueous uranyl acetate. Images were acquired using a ThermoFisher Talos L120C electron microscope equipped with a Ceta 16M camera, operated at 120 kV. The micrographs were acquired at 57,000× magnification (resulting in 2.26 Å per pixel) using EPU software. For 2D classification, 20,000 particles for ClpE WT, 13,641 particles for ClpE F375S, 10,000 particles for ClpE ΔN–E373A, and 5841 particles for ClpE C29A–C32A–E373A were selected using the boxing tool in EMAN2 (box pixel sizes 160 [ClpE WT, ClpE F375S], 120 [ClpE ΔN–E373A], or 200 [ClpE C29A–C32A–E373A]) (55). Image processing was carried out using the IMAGIC-4D package (56). Particles were band-pass filtered, normalized in their gray value distribution, and mass centered. 2D alignment, classification, and iterative refinement of class averages were performed as previously described (57).

ClpE (1 μM; WT or mutants) was preincubated for 15 min in the presence of 2 mM ATPγS at room temperature and diluted rapidly before application on the grid to a final concentration of 0.1 μM.

### Fluorescence microscopy

*E. coliΔclpB* cells harboring plasmids coding for *Sa* ClpP–S98A or *Sa* ClpP–WT and ClpE–YFP (WT and mutants) under an IPTG-controlled promoter were grown at 30 °C, and gene expression was induced by the addition of 25 μM IPTG (ClpE–YFP, ClpE–E373A–YFP) or 100 μM IPTG (ΔN–ClpE–YFP, ΔN–ClpE–E373A–YFP) for 90 min. For imaging, 1 ml culture was centrifuged at 13,000 rpm for 1 min in a tabletop centrifuge at room temperature, and the pellet was resuspended in 500 μl PBS buffer. The suspension (10 μl) was applied onto a 1% (w/v) agarose pad (in PBS buffer), which was then transferred onto a glass slide. Images were acquired using the Olympus CellSens widefield microscope and the 100x/1.49 oil immersion objective using YFP filters. Foci numbers and intensities were quantified by Fiji (https://imagej.net/software/fiji).

### *In vivo* toxicity assays (spot tests)

*E. coli ΔclpB* cells harboring plasmids coding for *Sa* ClpP and ClpE (WT and mutants) under an IPTG-controlled promoter were grown at 30 °C, 120 rpm overnight. Serial dilutions (10^−1^ to 10^−6^) after adjustment to an absorbance of at 600 nm were spotted on LB agar plates supplemented with different IPTG concentrations (0, 100, 250, and 500 μM) using a custom-made spotter (ZMBH workshop; University of Heidelberg). Plates were incubated overnight at 30/37/40 °C.

### Western blotting

Total cell extracts were prepared and separated *via* SDS-PAGE and subsequently electrotransferred onto a polyvinylidene fluoride membrane. Next, the membrane was incubated in blocking solution (3% bovine serum albumin [w/v] in Tris-buffered saline) for at least 30 min at room temperature. Protein levels were determined by incubating the membrane with ClpE-specific antibodies (1:10,000 dilution in Tris-buffered saline with Tween + 3% [w/v] bovine serum albumin) and an anti-rabbit alkaline phosphatase conjugate (Vector Laboratories) as a secondary antibody (1:20,000 dilution). YFP- and ClpP-specific antibodies were used at 1:10,000 and 1:5000 dilutions. Specificities of antibodies were documented in *E. coli* cells not expressing *Ef* ClpE, *Sa* ClpP, or YFP. Blots were developed using ECF Substrate (GE Healthcare) as a reagent and imaged using an Image-Reader LAS-4000 (Fujifilm).

### Cryo-EM sample vitrification and data collection

*Ef* ClpE–E221A/E373A/E551A and *Ef* ClpP (each 6 μM concentration) were incubated for 10 min in the presence of 3 μM concentration of casein at room temperature in a buffer containing 25 mM Tris–HCl (pH 7.5), 50 mM KCl, 20 mM MgCl_2_, 1 mM DTT, along with 2 mM ATP for complex formation. After incubation, 3 μl of the complex was applied to glow-discharged Quantifoil R2/2 carbon grid (Cu 300 mesh; STPLabtech) grids, which were treated for 60 s at 40 mA using a GloQube (Quorum) instrument. The grids were then plunged in liquid ethane for vitrification using a Vitrobot Mark IV (Thermo Fisher Scientific) set at 100% humidity and 24 °C, with a blot force of 1 and a blotting time of 2 s.

After vitrification, the grids were clipped, screened for ice thickness and sample concentration, and data were collected from the best-looking grid using a 300-kV Titan Krios G2 microscope (Thermo Fisher Scientific) equipped with a BioQuantum energy filter (slit at 20eV) and a K3 direct electron detector (Gatan/Ametek). A total of 14,937 micrographs were acquired at 105,000x nominal magnification, corresponding to a pixel size of 0.825 Å, with a total exposure dose of 40 e/Å^2^, and defocus values ranging from −2.2 to −0.5 μm with a step of 0.2 μm.

### Cryo-EM data processing

The process is summarized in Fig. S4.The cryoSPARC v5.0.2 (58) software package was used for patch motion correction and contrast transfer function estimation on all 14,937 micrographs and then curated based on average counts, contrast transfer function fit, and astigmatism to a total of 14,717 remaining good micrographs. Initially, a blob picker was used, tested both with a circular or an elliptical shape with a diameter range between 100 Å and 150 Å and an extraction box size of 500 pixels, which was subsequently binned down to 100 pixels for fast 2D classification. From ∼7.5 million initially blob-picked particles, 1,217,110 particles were selected based on visual inspection of 2D classes. These particles were re-extracted using a box size of 500 pixels, subsequently binned down to 256 pixels. Multiple rounds of 2D classification and *ab initio* volumes of generation facilitated classifying out noisy particles (junk), generating volumes of the ClpE–ClpP complex, including around 382,340 particles at ∼3.3 Å resolution (both with homogeneous and nonuniform refinement. From this particle set, more *ab initio* runs were performed to finally have enough different volumes, including fully formed Fc ClpE–ClpP complexes, mainly signal from ClpP and junk volumes, to carry out heterogeneous refinement to isolate the best particle subset to improve the complex map. The map, subsequent map, obtained from 159,660 particles, appeared slightly anisotropic, and an attempt of picking using Topaz was performed and resulted in ∼2 million particles picked from the original 14,717 micrographs. Upon cleaning with 2D classification and heterogeneous refinement, a good set of 556,680 particles was used to achieve a 2.88 Å resolution map of the *Ef* ClpE–ClpP complex. Extensive 3D classification with different subsets was performed and led to structure always around 3 Å resolution and displaying the same arrangement. Focused 3D classification of the ClpE ring, including also part of the apical ClpP barrel, was performed to investigate possible motion of the ClpE ring and the interface between ClpE and ClpP. Focused refinement of the ClpP part was performed, and after rebalancing the orientation distribution and applying D7 symmetry, an FcClpP map was obtained at 2.49 Å resolution.

### Model building

The initial monomeric atomic model of *Ef* ClpE was predicted using AlphaFold 3 (59, 60), both as monomer, dimers, or trimers. Single *Ef* ClpE chains were fitted into the best *Ef* ClpE–ClpP complex using chain refinement in Coot v0.89 (61, 62). The *Enterococcus faecium* ClpP tetradecamer crystal structure (Protein Data Bank ID: 6CFD) was used as the starting model for model building of the ClpP part in the ClpE–ClpP complex. This required the removal of the bound ADEP molecules in Protein Data Bank 6CFD and sequence changes. The combined initial model of *Ef* ClpE–ClpP complex was refined using several rounds of PHENIX v2.0 (63) real-space refinement alternated with Coot (61). The geometry of the fitted atomic model was improved using PHENIX geometry minimization and further refined first against the ClpE–ClpP EMready sharpened map and finally against the unsharpened map (statistics are reported in Table S3).

### Bioinformatic analyses

Multiple sequence alignments were performed using Clustal Omega (https://www.ebi.ac.uk/Tools/msa/clustalo/) and displayed using Jalview (www.jalview.org) (64). Phylogenetic analysis was adapted from the WoL: Reference Phylogeny for Microbes Project (https://biocore.github.io/wol/). Species were selected to create a representative overview of the bacterial kingdom. Protein occurrence was assigned based on correctly annotated sequences available from the UniProtKB database, and ambiguous entries were falsified *via* sequence length comparison and BLAST search. Statistical significance was tested using GraphPad Prism (GraphPad software). For multiple comparisons, we used ordinary one-way ANOVA with Dunnett’s multiple comparison test (Figs. 1*B*, 2, *B* and *C*, 4, *A* and *B*, 5, *D* and *E*, and S2, *A, B, D*, and *E*), and for unpaired comparison, we used the unpaired *t* test with Welch’s correction (Figs. 1*D*, 6, *F*, and S3 *D–F*, and *H–J*).

## Data availability

The *Ef* ClpE–ClpP cryo-EM density map, half maps, mask, Fourier shell correlation curves, and built models are being deposited into the Electron Microscopy Data Bank (https://www.ebi.ac.uk/pdbe/emdb/) under accession code D_1292154861. Aligned micrographs and raw frames are being deposited in EMPIAR. All the other data are contained within the article.

## Supporting information

This article contains supporting information (22, 65).

## Conflict of interest

The authors declare that they have no conflicts of interest with the contents of this article.

## Supporting information

DeRosa_SupplFigures_revrev_final

DeRosa_TableS1

De Rosa_Table S2

DeRosa_Table_S3
