# Supplementary material for: Structure–function analysis of the bacterial ClpE–ClpP AAA+ protease
Source: J Biol Chem. 2026 Mar 25;302(5):111403. doi: 10.1016/j.jbc.2026.111403 (PMC13125188; doi:10.1016/j.jbc.2026.111403)
Supplement: DeRosa_SupplFigures_revrev_final [file mmc1.pdf]

Supplementary Figure 1

Analysis *of Enterococcus faecalis* ClpE and *S.aureus* ClpP. (A/B) FITC-casein degradation and ATPase activities of ClpE were determined at indicated pH values. (C) LY-AMC degradation by Sa ClpP was determined at indicated pH values. (D) FITC-casein degradation rates were determined in absence of nucleotide (no nt) or in presence of ATPγS or an ATP regenerating system (ATP).

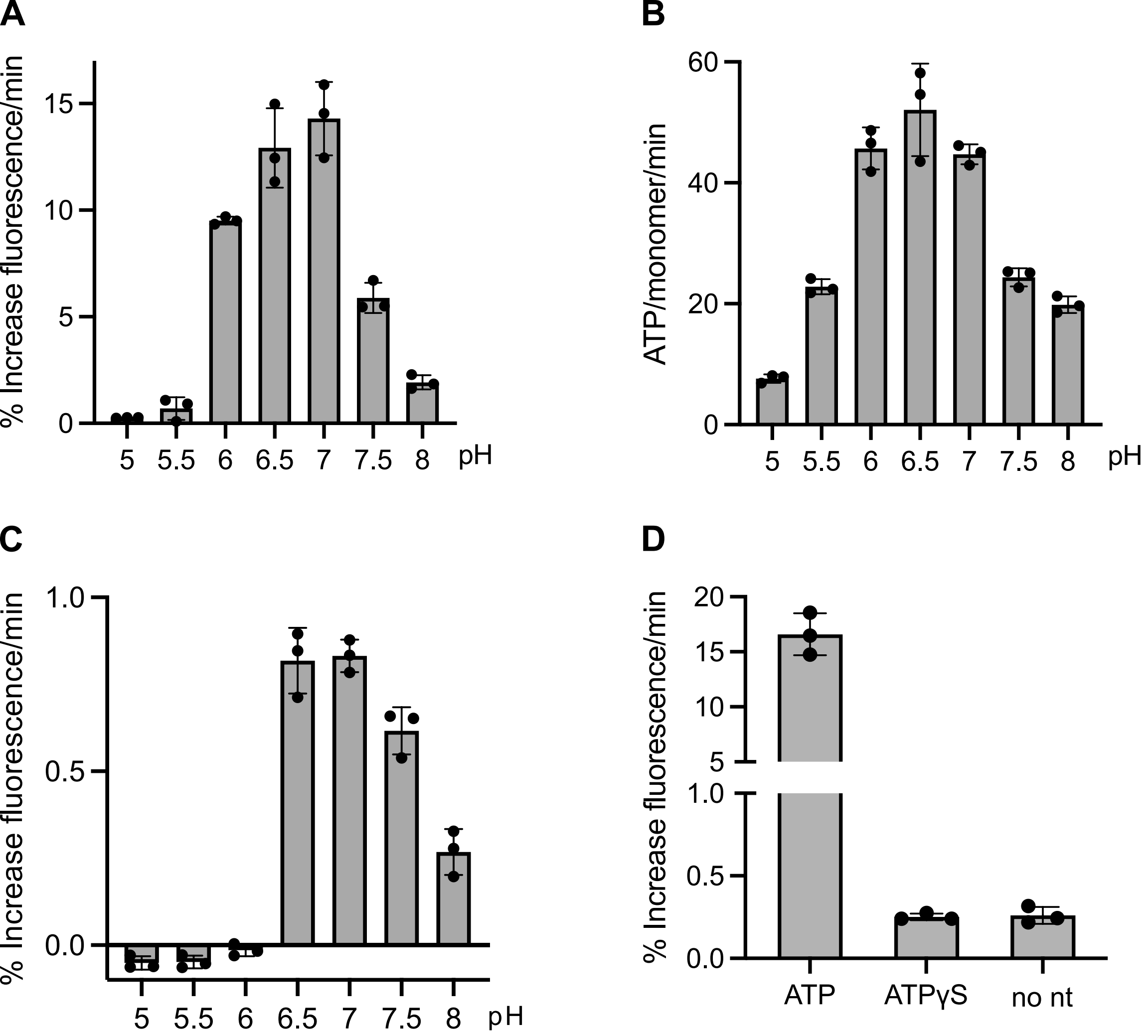
A
% Increase fluorescence/min
0
5
10
15
5
5.5
6
6.5
7
7.5
8
pH
B
ATP/monomer/min
0
20
40
60
5
5.5
6
6.5
7
7.5
8
pH
C
% Increase fluorescence/min
0.0
0.5
1.0
5
5.5
6
6.5
7
7.5
8
pH
D
% Increase fluorescence/min
0.0
0.5
1.0
5
10
15
20
ATP
ATPγS
no nt

Figure S1

Supplementary Figure 2

Comparison of ClpE and ClpC proteolytic activities. (A/B) Proteolytic activities of ClpE/ClpP and ClpC/ClpP (in absence or presence of the McsA/McsB and MecA adaptor proteins) were determined for the substrates FITC-casein and aggregated MDH. Degradation rates were determined based on changes (%/min) in FITC-casein fluorescence and MDH levels (via western blot analysis). The proteolytic activity of ClpE/ClpP was set to 1 for each substrate and the relative proteolytic activities of ClpC/ClpP are shown. The McsB-C270A mutant (McsB*) is deficient in kinase activity and MDH disaggregation. Error bars show standard deviations (n=3). (C) Refolding of aggregated MDH was determined at indicated timepoints in the presence of ClpE, ClpE/ClpP and ClpG. (D/E) MDH refolding rates (% reactivated MDH/min) and MDH refolding yields (% refolded MDH after 120 min) were determined. The activity of native MDH was set as 100%. Statistical analysis was performed by one-way ANOVA with Dunnett's multiple comparison test (A/B/D/E). ns: not significant, *: $p<0.05$, ***: $p<0.001$.****: $p<0.0001$.

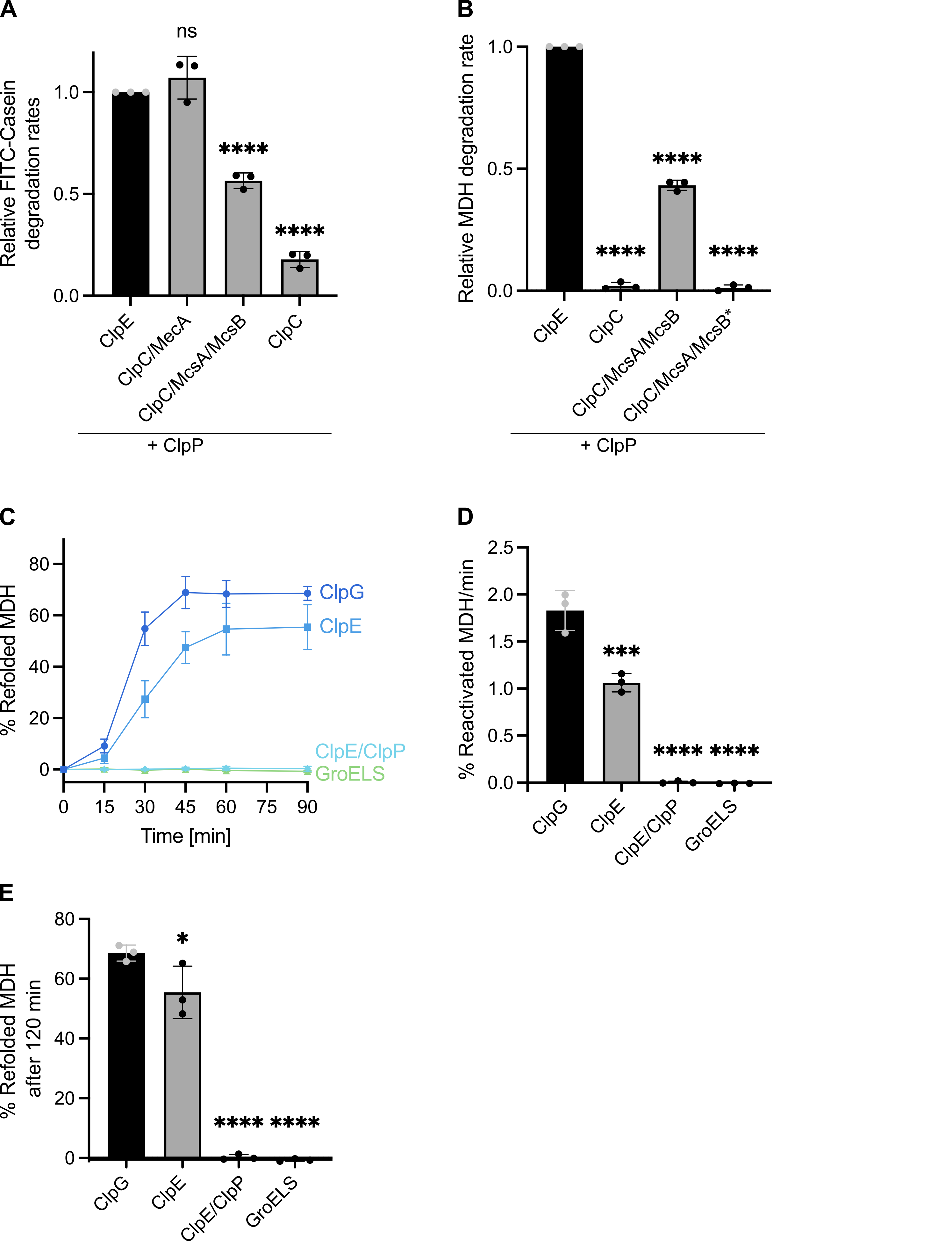

A
ns
Relative FITC-Casein degradation rates
1.0
0.5
0.0
****
****
ClpE
ClpC/MecA
ClpC/McsA/McsB
ClpC
+ ClpP
B
Relative MDH degradation rate
1.0
0.5
0.0
****
****
****
ClpE
ClpC
ClpC/McsA/McsB
ClpC/McsA/McsB*
+ ClpP
C
% Refolded MDH
80
60
40
20
0
ClpG
ClpE
ClpE/ClpP
GroELS
0
15
30
45
60
75
90
Time [min]
D
% Reactivated MDH/min
2.5
2.0
1.5
1.0
0.5
0.0
***
****
****
ClpG
ClpE
ClpE/ClpP
GroELS
E
% Refolded MDH after 120 min
80
60
40
20
0
*
****
****
ClpG
ClpE
ClpE/ClpP
GroELS

Figure S2

Supplementary Figure 3

Comparison of *S. aureus* and *E. faecalis* ClpP. (A) Sequence alignment of bacterial ClpPs. Conserved catalytic residues are boxed in red. Pronounced sequence alterations between *S. aureus* (Sa) and *E. faecalis* (Ef) ClpP are indicated with "*". (B) Degradation of the fluorescent peptide substrate LY-AMC by Sa or Ef ClpP was monitored in absence or presence of ADEP1. Initial LY-AMC fluorescence was set to 100. (C) FITC-casein degradation by ClpE complexed with either Sa or Ef ClpP. Initial FITC-casein fluorescence was set to 100. (D). Degradation of aggregated MDH by ClpE and Sa ClpP or Ef ClpP was determined by western blot analysis using MDH-specific antibodies. (E) Degradation of *B. subtilis* CtsR by ClpE and Sa ClpP or Ef ClpP was determined by SDS-PAGE analysis.

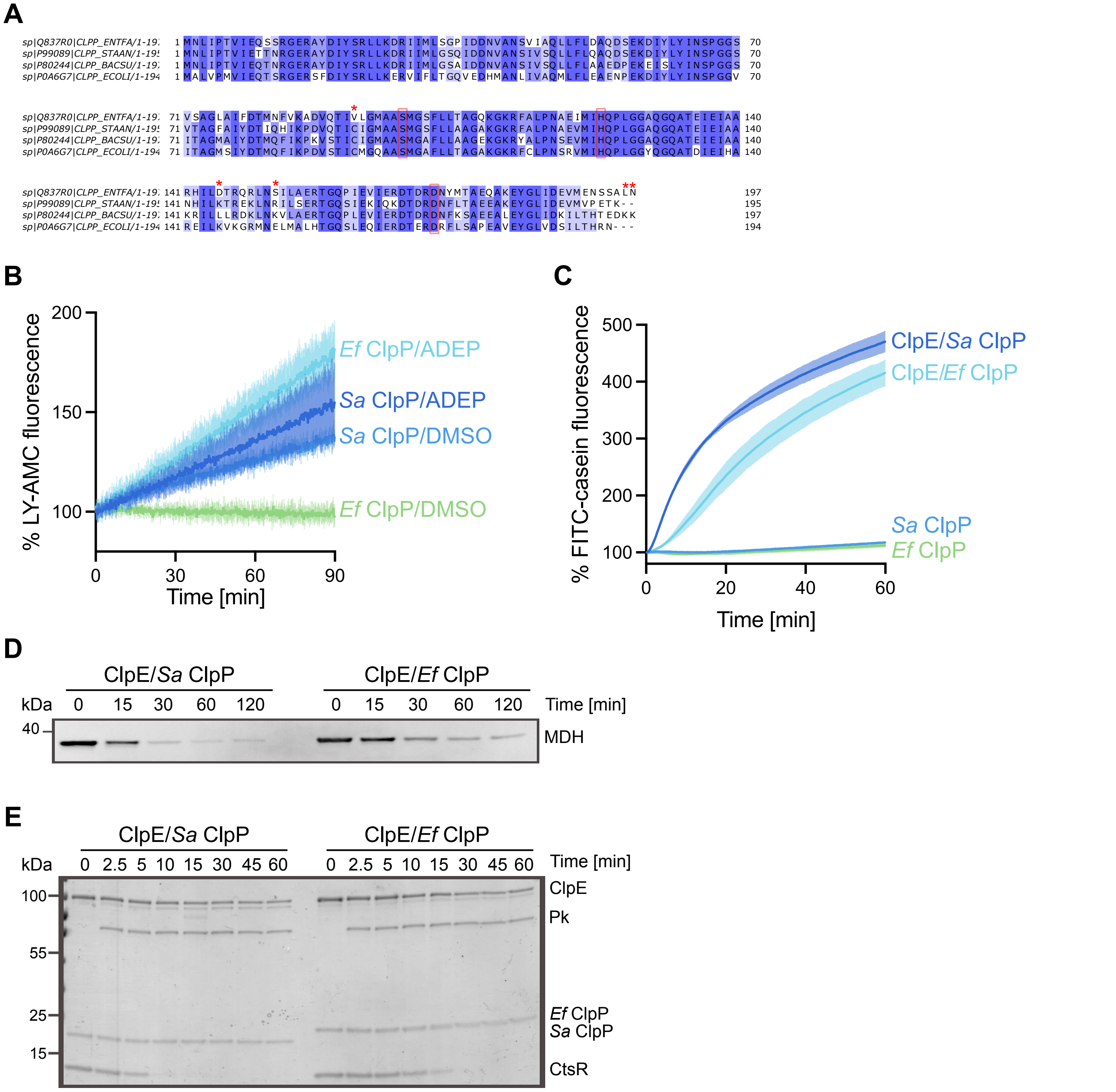
A
B
% LY-AMC fluorescence
Time [min]
Ef ClpP/ADEP
Sa ClpP/ADEP
Sa ClpP/DMSO
Ef ClpP/DMSO
C
% FITC-casein fluorescence
Time [min]
ClpE/Sa ClpP
ClpE/Ef ClpP
Sa ClpP
Ef ClpP
D
ClpE/Sa ClpP
ClpE/Ef ClpP
kDa
Time [min]
MDH
E
ClpE/Sa ClpP
ClpE/Ef ClpP
kDa
Time [min]
ClpE
Pk
Ef ClpP
Sa ClpP
CtsR

Figure S3

Supplementary Figure 4

Cryo-EM processing workflow of the *Enterococcus faecalis* ClpE/ClpP dataset. Example of micrographs of vitrified complex, particle picking strategy (both with blob picker and with Topaz, 2D classification and subsequent runs of 3D ab initio reconstruction, 3D heterogeneous refinement, 3D classification and homogeneous refinement with sharpening are given. A full description of the processing pathways is also given in the Material and Methods session.

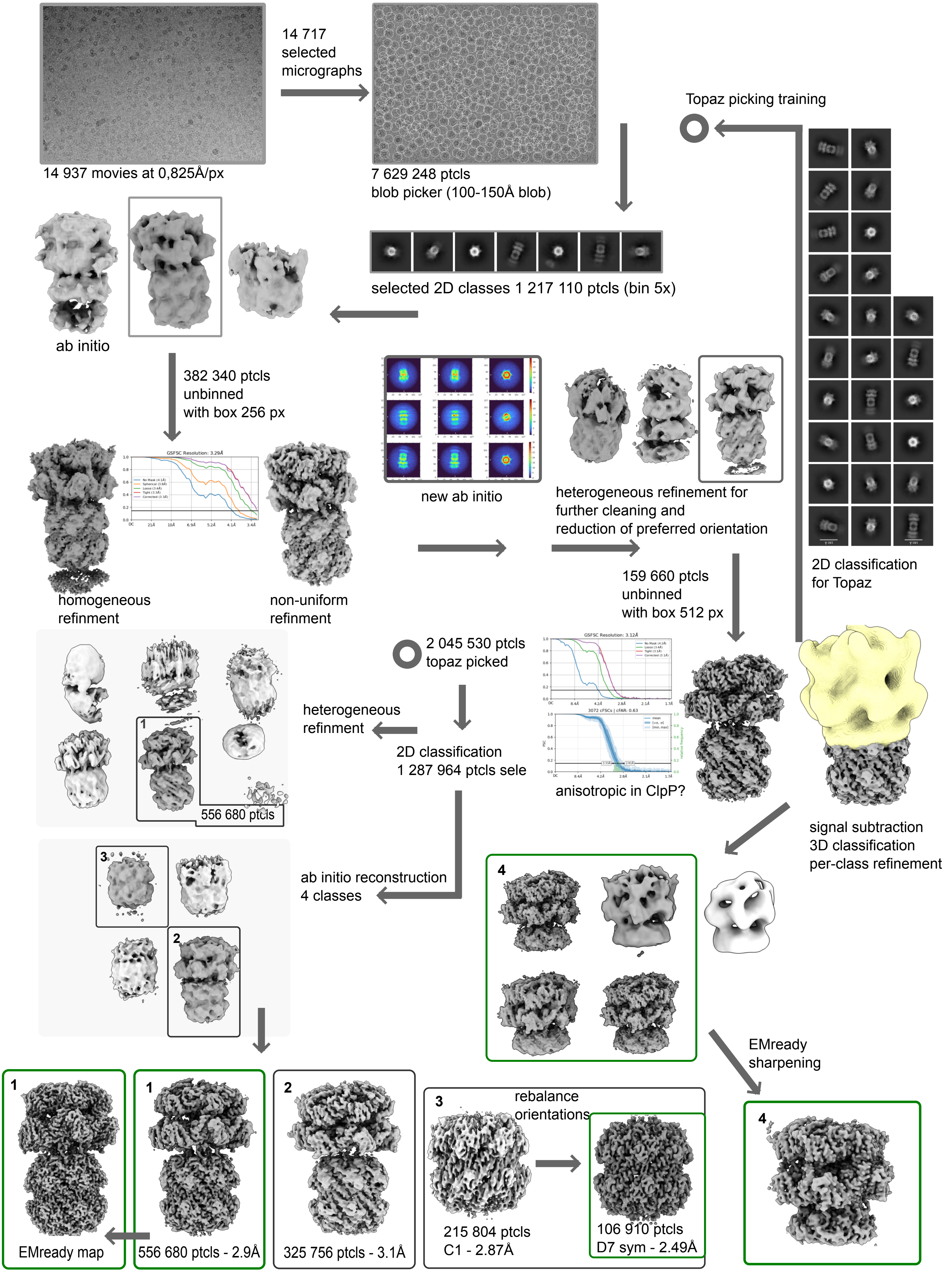
14 937 movies at 0,825Å/px
14 717
selected
micrographs
7 629 248 ptcls
blob picker (100-150Å blob)
Topaz picking training
selected 2D classes 1 217 110 ptcls (bin 5x)
ab initio
382 340 ptcls
unbinned
with box 256 px
GSFSC Resolution: 3.29Å
homogeneous
refinment
non-uniform
refinment
new ab initio
heterogeneous refinement for
further cleaning and
reduction of preferred orientation
2D classification
for Topaz
159 660 ptcls
unbinned
with box 512 px
GSFSC Resolution: 3.12Å
3072 cFSCs | cFAR: 0.63
anisotropic in ClpP?
2 045 530 ptcls
topaz picked
heterogeneous
refinment
2D classification
1 287 964 ptcls sele
1
556 680 ptcls
signal subtraction
3D classification
per-class refinement
ab initio reconstruction
4 classes
3
2
4
EMready
sharpening
1
EMready map
1
556 680 ptcls - 2.9Å
2
325 756 ptcls - 3.1Å
3
rebalance
orientations
215 804 ptcls
C1 - 2.87Å
106 910 ptcls
D7 sym - 2.49Å
4

Figure S4

Supplementary Figure 5

Comparison of *S. aureus* ClpC/ClpP and *E. faecalis* ClpE/ClpP complexes. (A) Superimposition of the Ef ClpP barrel with Sa ClpP shows almost perfect agreement. On the left superimposition of the atomic models displayed as ribbons and on the right of the corresponding maps. On the lower raw a cut through of the model and maps is displayed to show the inside of the proteolytic chamber. On the right, in the model fitted into the map, the catalytic residues for both Ef (in black) and Sa (in magenta) ClpP residues are highlighted. The zoomed inset shows details of the active centre. (B) Overlapping of the Ef ClpE/ClpP model (in grey) with the Sa ClpC/ClpP (in magenta) model shows very similar organisation. (C) Nucleotide-binding state of the ClpE ATPase subunits both in the AAA1, upper raw, and in the AAA2, lower raw, rings. The subunits are shown as ribbon liquorice and the nucleotides are shown as sticks coloured by element, the R-fingers of a neighboring subunits contacting ATP are shown in magenta sticks.

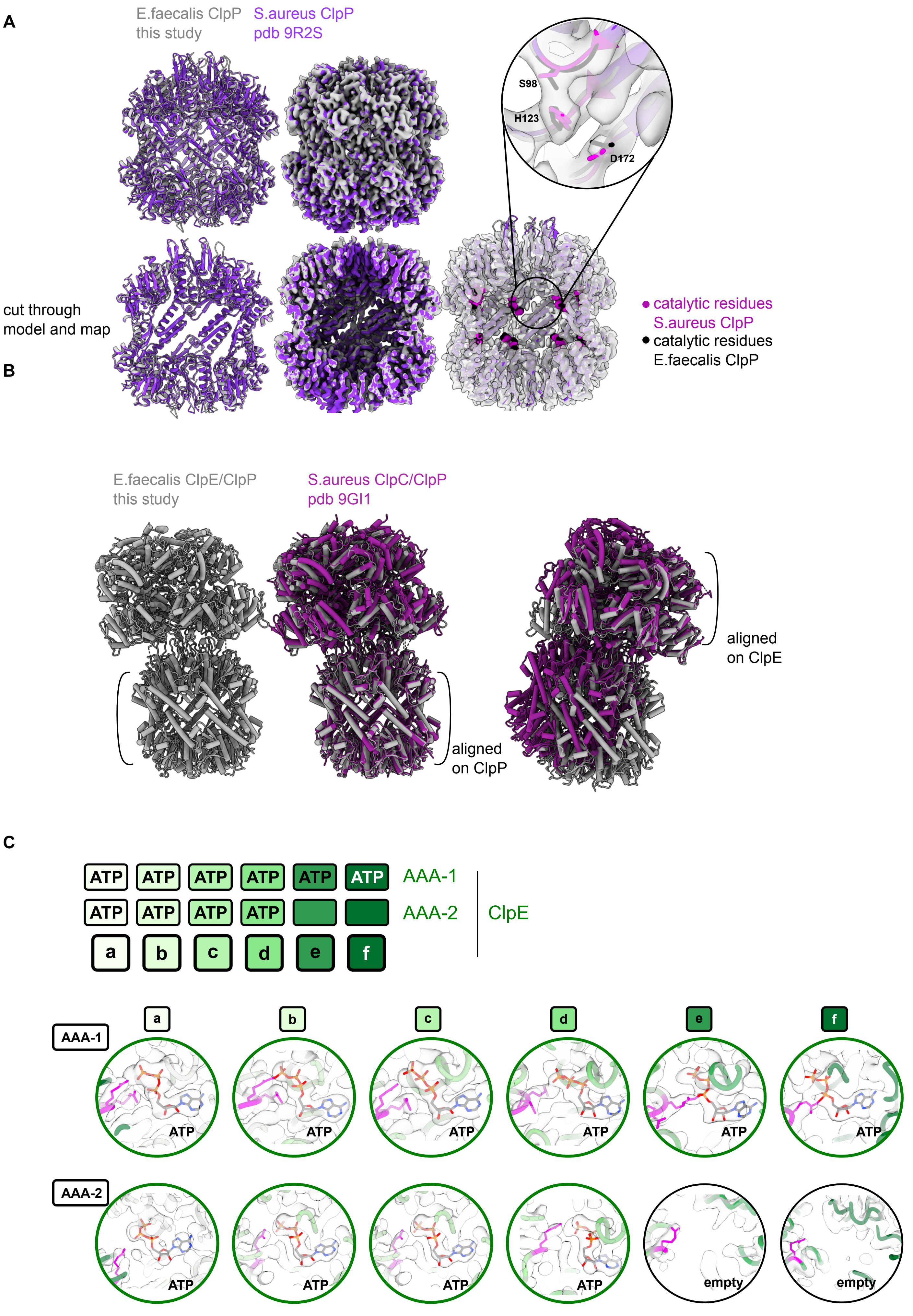
A
E.faecalis ClpP
this study
S.aureus ClpP
pdb 9R2S
S98
H123
D172
cut through
model and map
catalytic residues
S.aureus ClpP
catalytic residues
E.faecalis ClpP
B
E.faecalis ClpE/ClpP
this study
S.aureus ClpC/ClpP
pdb 9GI1
aligned
on ClpE
aligned
on ClpP
C
ATP ATP ATP ATP ATP ATP AAA-1
ATP ATP ATP ATP AAA-2
ClpE
a b c d e f
AAA-1
AAA-2
ATP
empty

Figure S5

Supplementary Figure 6

Oligomerization of ClpE. (A) Models of reported Hsp100 assemblies. ClpC forms an inactive decameric resting state, while *M. tuberculosis* ClpC1 bound to Cyclomarin A (CymA) forms a tetrahedron structure composed of four hexamers. A similar structure has been described for *L. monocytogenes* ClpL, which additionally forms ring dimers and single rings. MecA-activated ClpC forms single rings hexamers. (B) Hydrodynamic radii of ClpE wild-type (wt) and MD-mutants E373A and F375S were determined by DLS measurements in absence and presence of ClpP. Boxes represent the interquartile range (IQR), which spans from the first quartile (Q1) to the Q3. The length of the box indicates the spread of the middle 50% of the data. Whiskers extend from the minimum and maximum values within 1.5× IQR. The median is indicated by the horizontal line. (C) Glutaraldehyde (GA) crosslinking of ClpE wt and indicated MD mutants was performed in presence of ATPγS. Crosslinking reactions were analyzed before (0 min) and after (10 min) GA addition by SDS-PAGE. Crosslink product identities are indicated. A protein standard (kDa) is provided. (D) Particle size distributions (% frequency) of DLS data were determined for ClpE wt and the MD mutants E373A and F375S in presence of *E. faecalis* ClpP. The hydrodynamic radius of the most populated state is indicated. Standard deviations (n=30) are shown as shaded area. (E/F) Gallery of representative 2D class averages of ClpE wt and ClpE-F375S. Scale bar = 10 nm.

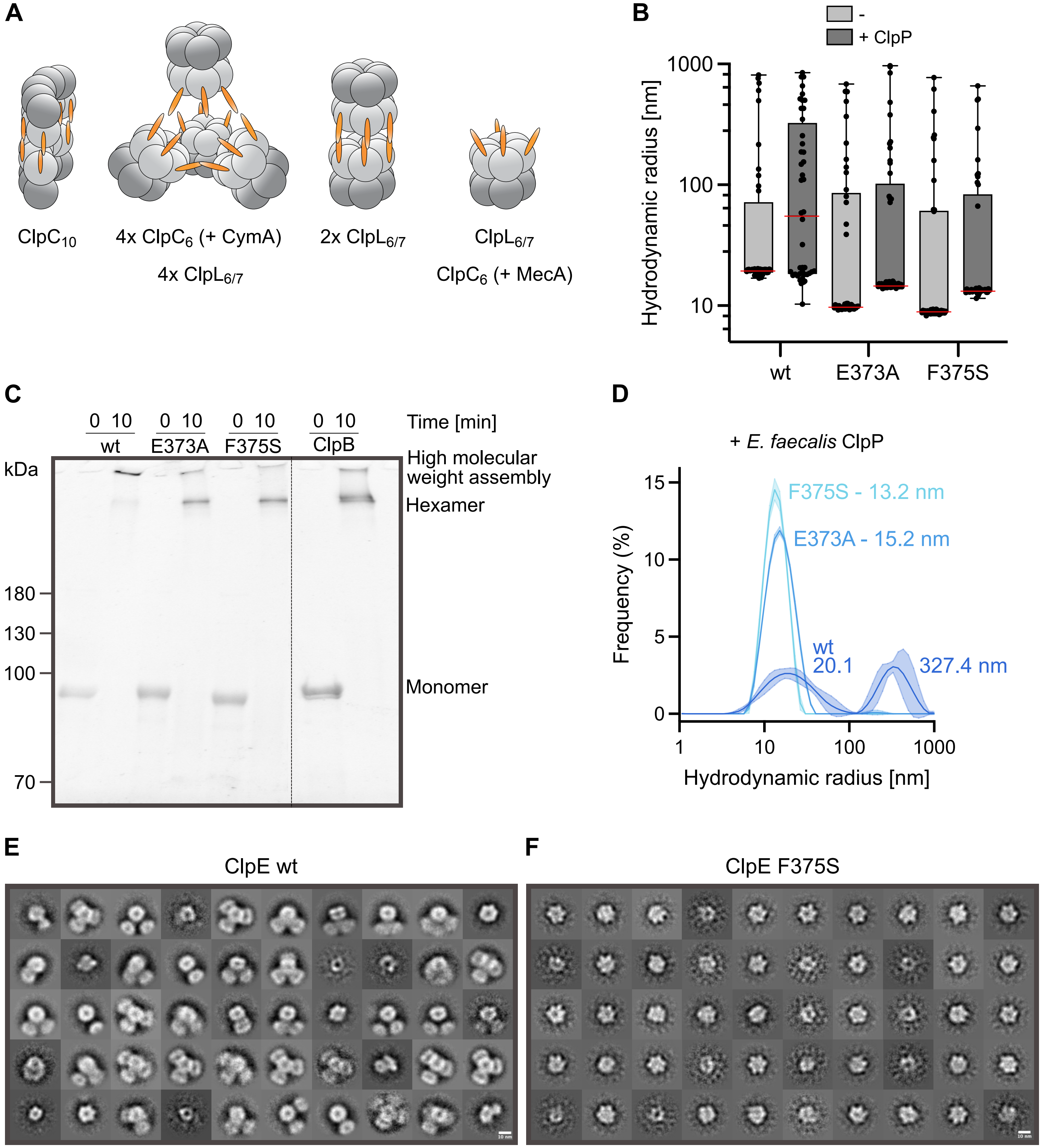

A
ClpC10
4x ClpC6 (+ CymA)
4x ClpL6/7
2x ClpL6/7
ClpL6/7
ClpC6 (+ MecA)
B
-
+ ClpP
Hydrodynamic radius [nm]
1000
100
10
wt
E373A
F375S
C
Time [min]
0 10
wt
0 10
E373A
0 10
F375S
0 10
ClpB
kDa
180
130
100
70
High molecular weight assembly
Hexamer
Monomer
D
+ E. faecalis ClpP
F375S - 13.2 nm
E373A - 15.2 nm
wt
20.1
327.4 nm
Frequency (%)
15
10
5
0
1
10
100
1000
Hydrodynamic radius [nm]
E
ClpE wt
F
ClpE F375S

Figure S6

Supplementary Figure 7

Characterization of ClpE NTD mutants. (A) Domain organizations and sequence alignment of the ClpG N1-domain and ClpE N-domain. Conserved residues involved in Zn^{2+}-binding are boxed in red. The NMR structure of the ClpG N1 domain (PDB ID 8P66, residues 1-46) and the AlphaFold2 model of the ClpE N-domain (residues 1-46) are shown. (B) Particle size distributions (% frequency) of DLS data were determined for indicated N-domain mutant derivatives of ClpE-E373A in absence (top) and presence (bottom) of Sa ClpP. The hydrodynamic radius of the most populated state is indicated. Standard deviations (n=30) are shown as shaded area. (C) Hydrodynamic radii of ClpE-E373A and mutant derivatives were determined by DLS in absence and presence of Sa ClpP. Data are represented as described in Supplementary Figure 6B. (D) 2D class averages of ΔN-ClpE-E373A based on negative stain EM. Scale bar = 10 nm. (E) FITC-casein binds to the N-terminal domain of ClpE. Binding of ClpE-E373A, ΔN-ClpE-E373A and ΔN_N-ClpE-E373A to FITC-casein was monitored in presence of ATPγS and increasing ClpE concentrations by determining changes in FITC-casein anisotropy. Standard deviations are shown (n=2). (F) Degradation rates of aggregated MDH by ClpE-E373A and Sa ClpP or Ef ClpP were determined by turbidity measurements. The proteolytic activity of ClpE-E373A/Sa ClpP was set to 1. (G) Proteolytic activities of indicated ClpE-E373A NTD mutants in complex with Ef ClpP. MDH disaggregation rates were determined and the activity of ClpE-E373A/Ef ClpP was set to 1. (H) Reactivation of aggregated MDH by ClpE-E373A NTD mutants. MDH refolding rates (left) and yields (right) were determined and the refolding activity of ClpE-E373A was set to 1. Statistical analysis was performed by one-way ANOVA with Dunnett's multiple comparison test (F-H). ns: not significant, *: $p<0.05$, ***: $p<0.001$.****: $p<0.0001$.

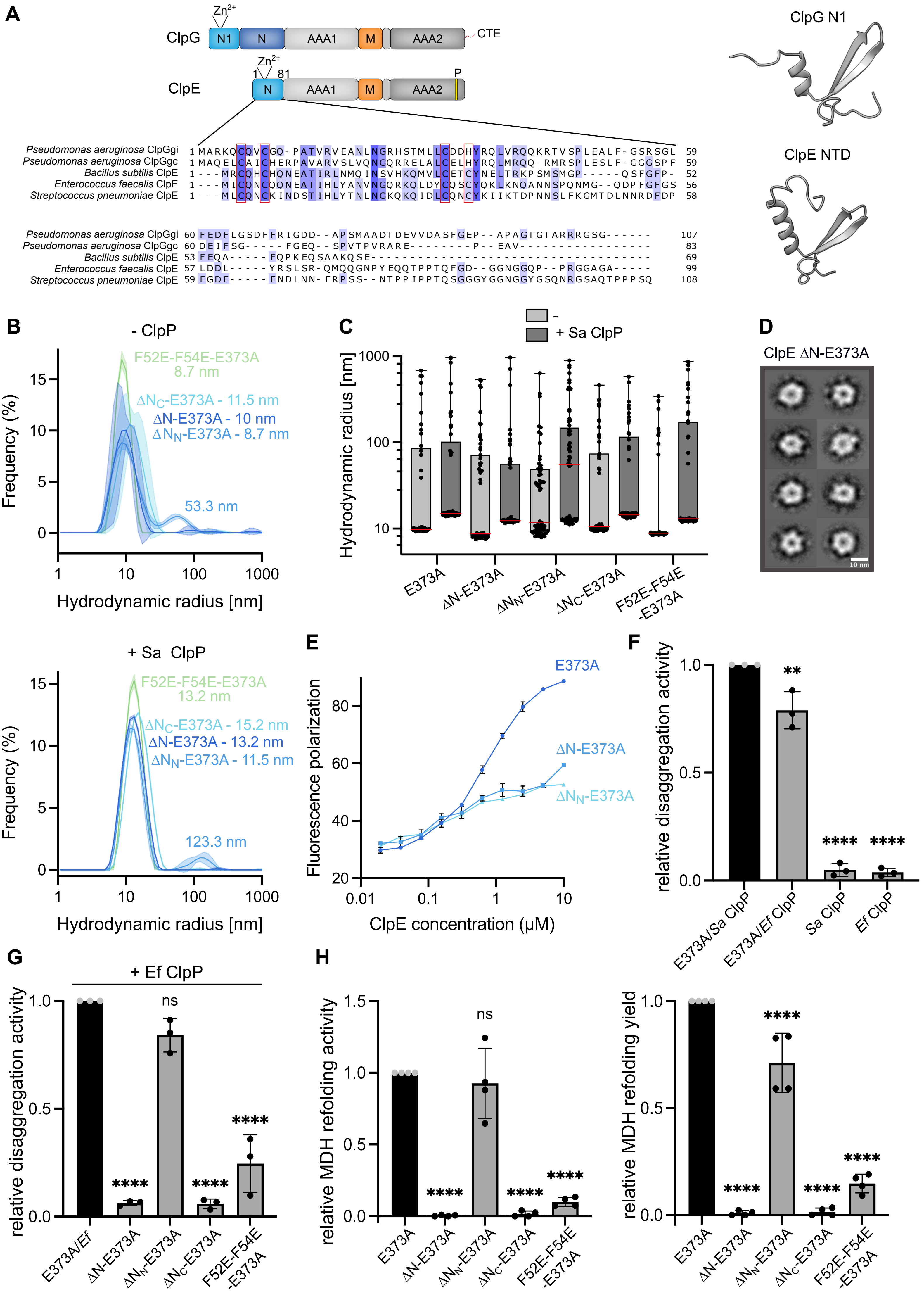

A
ClpG
Zn2+
N1
N
AAA1
M
AAA2
CTE
ClpE
1
81
P
ClpG N1
ClpE NTD
Pseudomonas aeruginosa ClpGgi 1 MARKQCQVCGQ-PATVRVEANLNGRHSTMLLCDDHYRQLVRQQKRTVSPLEALF-GSRSGL 59
Pseudomonas aeruginosa ClpGgc 1 MAQELCAICHERPAVARVSLVQNGQRRELALCELHYRQLMRQQ-RMRSPLESLF-GGGSPF 59
Bacillus subtilis ClpE 1 ---MRCQHCHQNEATIRLNMQINSVHKQMVLCETCYNELTRKPSMSMGP-----QSFGFP- 52
Enterococcus faecalis ClpE 1 ---MICQNCQQNEATIHLYANVNGRQKQLDYCQSCYQKLKNQANNSPQNMG--QDPFGFGS 56
Streptococcus pneumoniae ClpE 1 ---MLCQNCKINDSTIHLYTNLNGKQKQIDLCQNCYKIIKTDPNNSLFKGMTDLNNRDFDP 58
Pseudomonas aeruginosa ClpGgi 60 FEDFLGSDFFRIGDD--APSMAADTDEVVDASFGEP--APAGTGTARRRGSG------ 107
Pseudomonas aeruginosa ClpGgc 60 DEIFSG-----FGEQ--SPVTPVRARE--------P--EAV----------------- 83
Bacillus subtilis ClpE 53 FEQA-----FQPKEQSAAKQSE------------------------------------- 69
Enterococcus faecalis ClpE 57 LDDL-----YRSLSR-QMQQGNPYEQQTPPTQFGD--GGNGGQP--PRGGAGA---- 99
Streptococcus pneumoniae ClpE 59 FGDF-----FNDLNN-FRPSS--NTPPIPPTQSGGGYGGNGGYGSQNRGSAQTPPPSQ 108
B
- ClpP
F52E-F54E-E373A
8.7 nm
ΔNC-E373A - 11.5 nm
ΔN-E373A - 10 nm
ΔNN-E373A - 8.7 nm
53.3 nm
Frequency (%)
Hydrodynamic radius [nm]
+ Sa ClpP
F52E-F54E-E373A
13.2 nm
ΔNC-E373A - 15.2 nm
ΔN-E373A - 13.2 nm
ΔNN-E373A - 11.5 nm
123.3 nm
C
-
+ Sa ClpP
Hydrodynamic radius [nm]
E373A
ΔN-E373A
ΔNN-E373A
ΔNC-E373A
F52E-F54E -E373A
D
ClpE ΔN-E373A
10 nm
E
E373A
ΔN-E373A
ΔNN-E373A
Fluorescence polarization
ClpE concentration (µM)
F
relative disaggregation activity
E373A/Sa ClpP
E373A/Ef ClpP
Sa ClpP
Ef ClpP
G
+ Ef ClpP
relative disaggregation activity
ns
E373A/Ef
ΔN-E373A
ΔNN-E373A
ΔNC-E373A
F52E-F54E -E373A
H
relative MDH refolding activity
relative MDH refolding yield
E373A

Supplementary Figure 8

Impact of Zn^{2+}-binding on ClpE assembly and activity. (A) Autoprocessing of ClpE wt, E373A and C29A-C32A-E373A was monitored in presence of substrate CtsR by SDS-PAGE and Coomassie staining. The positions of full-length and cleaved ClpE are indicated. (B) Band intensities of full-length and cleaved ClpE and CtsR were quantified and the cleavage/degradation kinetics and efficiencies were calculated (n=3). (C) Bands corresponding to full-length and cleaved ClpE were excised from SDS-gel and analyzed by mass spectrometry. Peptides that were identified for both bands are shown in yellow, peptides that were only identified for full-length ClpE are shown in green. (D) Cryo-EM structure of the ClpA/ClpP complex (PDB-ID 6UQO), as model for AAA+ protease organization. The distance between the entry of the AAA+ translocation channel and the proteolytic active sites of ClpP (shown in red) is approx. 140Å, corresponding to approx. 40 residues (aa). (E) Particle size distributions (% frequency) of DLS data were determined for ClpE-C29A-C32A-E373A in absence and presence of Sa ClpP. The hydrodynamic radii of the most populated states are indicated. Standard deviations (n=30) are shown as shaded areas. (F) Hydrodynamic radii of ClpE-E373A and ClpE-C29A-C32A-E373A were determined by DLS in absence and presence of Sa ClpP. Data are represented as described in Supplementary Figure 6B. (G) Gallery of representative 2D class averages of ClpE-C29A-C32A-E373A. Scale bar = 20 nm.

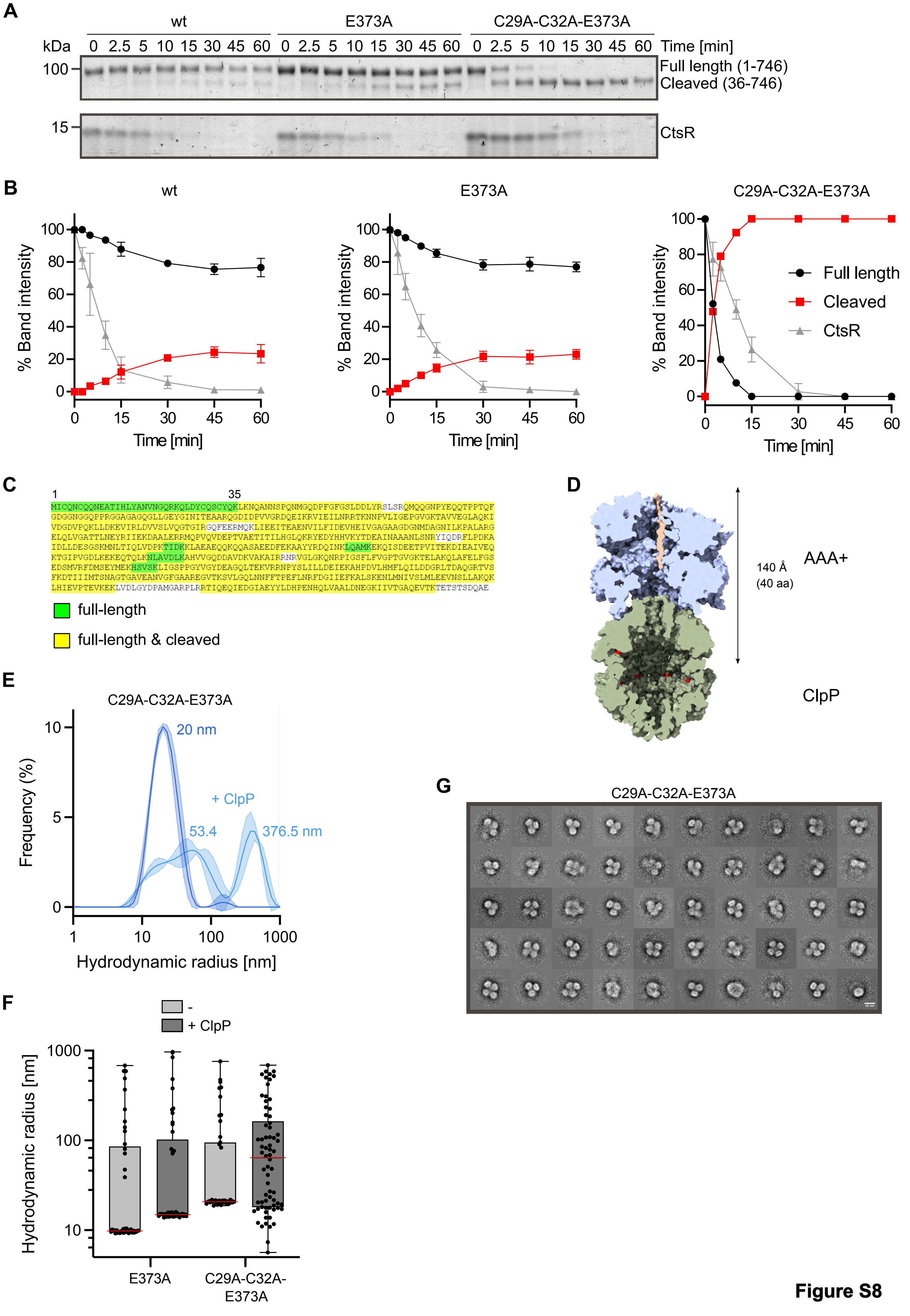
A
wt
E373A
C29A-C32A-E373A
kDa
0 2.5 5 10 15 30 45 60
Time [min]
100
Full length (1-746)
Cleaved (36-746)
15
CtsR
B
% Band intensity
Time [min]
Full length
Cleaved
CtsR
C
1
35
full-length
full-length & cleaved
D
140 Å
(40 aa)
AAA+
ClpP
E
C29A-C32A-E373A
20 nm
+ ClpP
53.4
376.5 nm
Frequency (%)
Hydrodynamic radius [nm]
F
-
+ ClpP
Hydrodynamic radius [nm]
E373A
C29A-C32A-E373A
G
C29A-C32A-E373A

Figure S8

Supplementary Figure 9

ClpE/ClpP expression creates toxicity in *E. coli*. (A) *E. coli* cells expressing the indicated plasmid-encoded *clp* alleles and harboring either pDMI.1 (top) or pDMI.1-*clpP* (bottom) were grown overnight at 30°C and adjusted to an OD_{600} of 1. Serial dilutions (10^{-1} – 10^{-6}) were spotted on LB plates containing the indicated IPTG concentrations and incubated at 30 °C or 40 °C for 24 h. p = vector control. (B) Degradation of FITC-casein by ClpE was monitored in presence of *E. coli* ClpP or *S. aureus* ClpP. A reaction including *E. coli* ClpA and *E. coli* ClpP served as positive control. (C) Total lysates were prepared from colonies (from LB plates incubated at 30°C and including 50 μM IPTG) and analyzed by SDS-PAGE and Coomassie staining. The positions of ClpE wt and mutants and ClpP are indicated. A protein standard (kDa) is provided. (D) *E. coli* cells expressing the indicated plasmid-encoded *clpE-yfp* alleles and harboring either pDMI.1 (top) or pDMI.1-*clpP* (bottom) were grown overnight at 30°C and adjusted to an OD_{600} of 1. Serial dilutions (10^{-1} – 10^{-6}) were spotted on LB plates containing the indicated IPTG concentrations and incubated at 30 °C or 40 °C for 24 h. p = vector control. (E) *E. coli* cells expressing the indicated plasmid-encoded *clpE* alleles and harboring pDMI.1-*clpP* were grown overnight at 30°C. Total lysates were prepared and analyzed by SDS-PAGE and Coomassie staining. The positions of ClpE wt and mutants and ClpP are indicated. A protein standard (kDa) is provided. (F) Total lysates were prepared from colonies (from LB plates incubated at 30°C and including 50 μM IPTG) and analyzed by SDS-PAGE and Coomassie staining. The position of ClpE-YFP (wt and mutants) and ClpP are indicated. A protein standard (kDa) is provided. ClpE-YFP levels were additionally monitored by western blot analysis using YFP-specific antibodies.

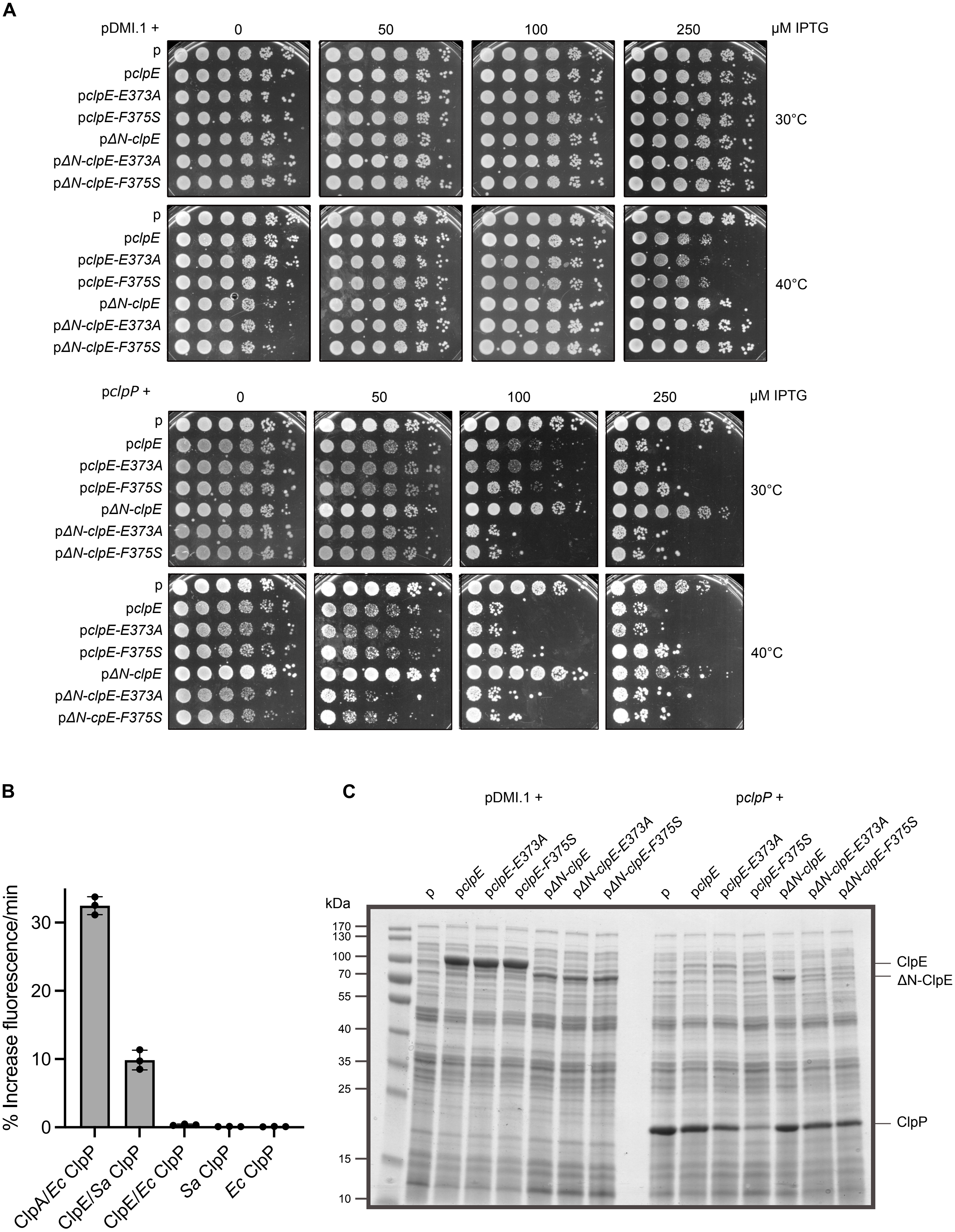
A
pDMI.1 +
0
50
100
250
μM IPTG
p
pclpE
pclpE-E373A
pclpE-F375S
pΔN-clpE
pΔN-clpE-E373A
pΔN-clpE-F375S
30°C
40°C
pclpP +
pΔN-cpE-F375S
B
% Increase fluorescence/min
0
10
20
30
ClpA/Ec ClpP
ClpE/Sa ClpP
ClpE/Ec ClpP
Sa ClpP
Ec ClpP
C
pDMI.1 +
pclpP +
kDa
170
130
100
70
55
40
35
25
15
10
ClpE
ΔN-ClpE
ClpP

Figure S9

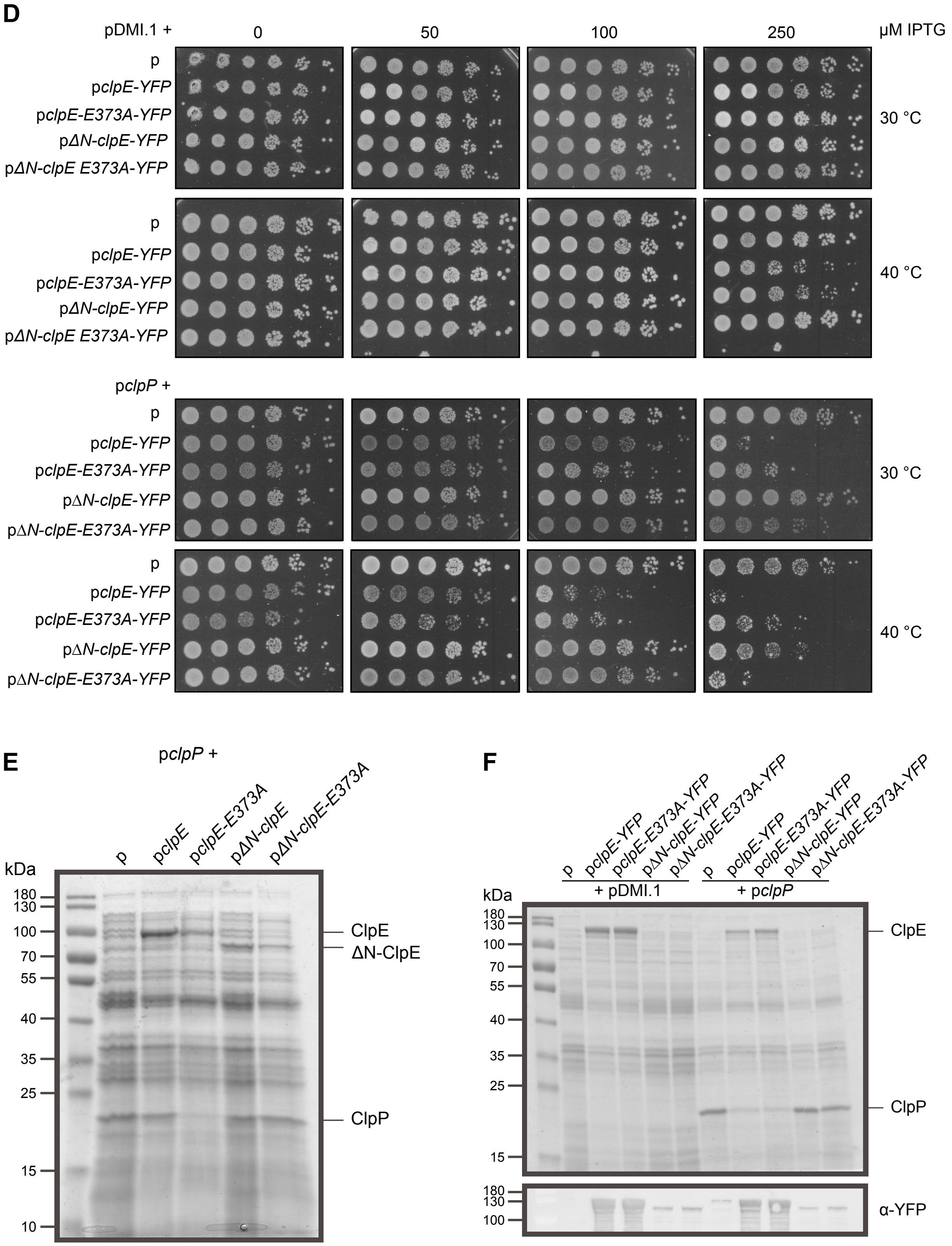
D
pDMI.1 +
0
50
100
250
µM IPTG
p
pclpE-YFP
pclpE-E373A-YFP
pΔN-clpE-YFP
pΔN-clpE E373A-YFP
30 °C
40 °C
pclpP +
pΔN-clpE-E373A-YFP
E
pclpP +
p
pclpE
pclpE-E373A
pΔN-clpE
pΔN-clpE-E373A
kDa
180
130
100
70
55
40
35
25
15
10
ClpE
ΔN-ClpE
ClpP
F
+ pDMI.1
+ pclpP
α-YFP

Figure S9

Supplementary Figure 10

(A) *E. coli* cells expressing the indicated plasmid-encoded *clpE-yfp* alleles and harboring either pDMI.1 (vc), p*clpP* (P) or p*clpP-S98A* (P*) were grown to mid-exponential growth phase at 30 °C in presence of 25 µM IPTG (p*clpE*, p*clpE-VGF**, p*clpE-E373A,* p: empty vector) or 100 µM *(*p*ΔN-clpE,* p*ΔN-clpE-E373A*) IPTG. Total cell lysates were prepared and analyzed by SDS-PAGE. A protein standard (kDa) is indicated. ClpE-YFP (wt and mutants) and ClpP levels were determined by western blot analysis using ClpE- and YFP-specific or ClpP-specific antibodies. (B) *E. coli* cells expressing the indicated plasmid-encoded *clpE-yfp* alleles and harboring either pDMI.1 (p), p*clpP* or p*clpP-S98A* were grown overnight at 30°C and adjusted to an OD_{600} of 1. Serial dilutions (10^{-1} – 10^{-6}) were spotted on LB plates containing the indicated IPTG concentrations and incubated at 30 °C or 40 °C for 24 h. (C) Total lysates were prepared from colonies (from LB plates incubated at 30°C and including 50 µM IPTG) and analyzed by SDS-PAGE and Coomassie staining. The positions of ClpE-YFP (wt and mutants) and ClpP are indicated. (D) Cellular localization of ClpE-YFP and ClpE-VGF*-YFP fusions in *E. coli* cells. *E. coli* cells expressing the indicated plasmid-encoded *clpE-yfp* alleles under control of an IPTG-regulatable promoter and harboring p*clpP* were grown at 30°C in presence of 25 µM IPTG. ClpE-YFP localizations were determined by fluorescence microscopy. Scale bar: 5 µm. Boxed cells are additionally shown as enlarged image. (E) Percentage of cells harboring the indicated number of ClpE-YFP and ClpE-VGF*-YFP foci were determined in presence of ClpP (n>80, two independent biological replicates). (F) Relative intensities of ClpE-YFP and ClpE-VGF*-YFP foci (% of total cellular fluorescence) were determined in presence of ClpP (n>25, two independent biological replicates). Statistical Analysis: Welch's unpaired t-test. Significance levels: ****: $p < 0.0001$.

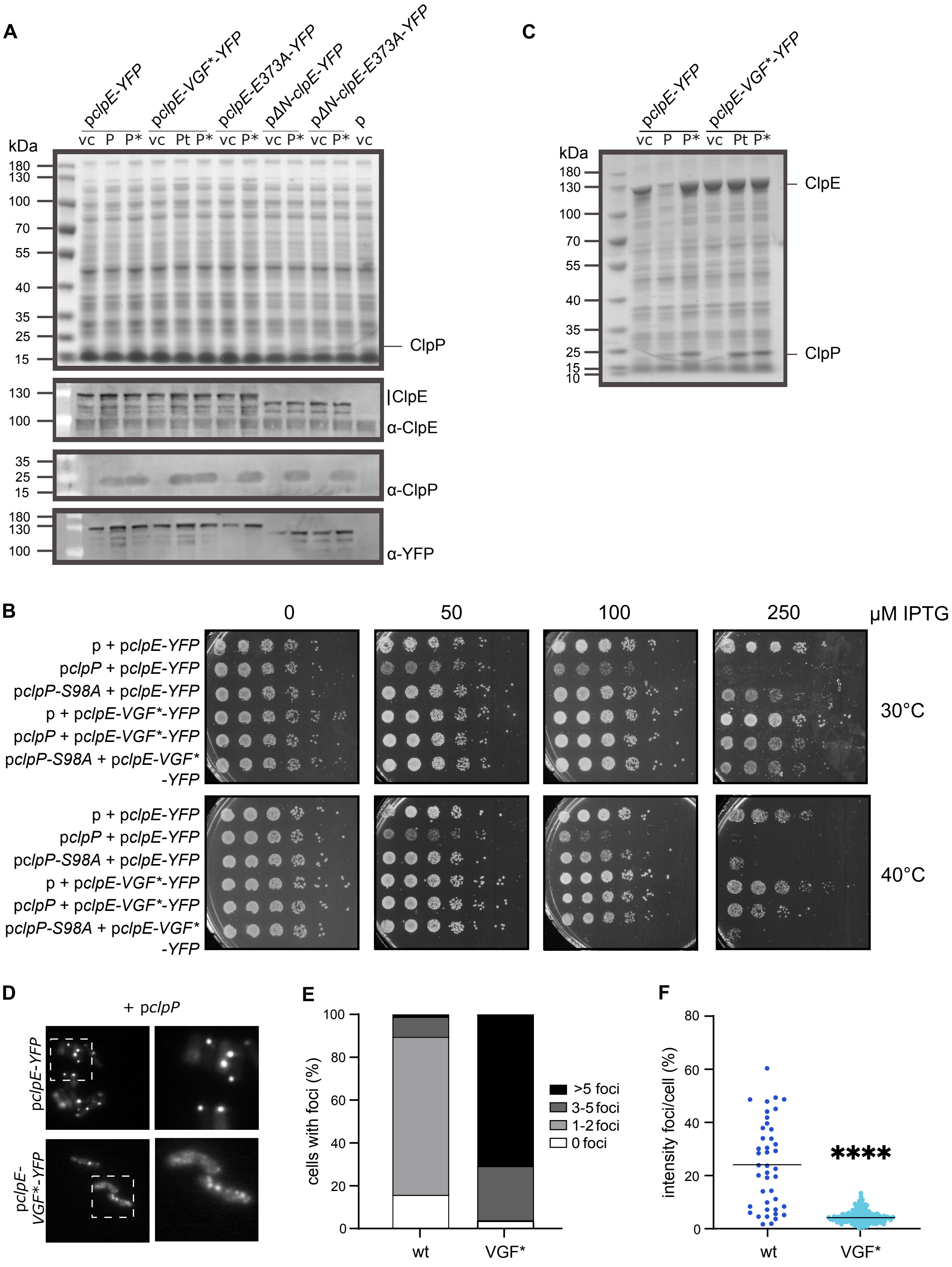
A
pclpE-YFP
pclpE-VGF*-YFP
pclpE-E373A-YFP
pΔN-clpE-YFP
pΔN-clpE-E373A-YFP
p
vc P P* vc Pt P* vc P* vc P* vc P* vc
kDa
ClpP
ClpE
α-ClpE
α-ClpP
α-YFP
B
0
50
100
250
μM IPTG
p + pclpE-YFP
pclpP + pclpE-YFP
pclpP-S98A + pclpE-YFP
p + pclpE-VGF*-YFP
pclpP + pclpE-VGF*-YFP
pclpP-S98A + pclpE-VGF*-YFP
30°C
40°C
C
pclpE-YFP
pclpE-VGF*-YFP
vc P P* vc Pt P*
ClpE
ClpP
D
+ pclpP
pclpE-YFP
pclpE-VGF*-YFP
E
cells with foci (%)
>5 foci
3-5 foci
1-2 foci
0 foci
wt
VGF*
F
intensity foci/cell (%)
****
wt
VGF*

Figure S10

Supplementary Figure 11

Phylogenetic tree showing the occurrence of ClpB, ClpC, ClpE and ClpL in representative bacterial species. Selected classes are indicated with colored panels. The tree was constructed with data from https://biocore.github.io/wol/. Protein occurrence was determined by matching source organisms from unambiguously annotated UniProtKB database entries for ClpB, ClpC, ClpE and ClpL with the phylogenetic data.

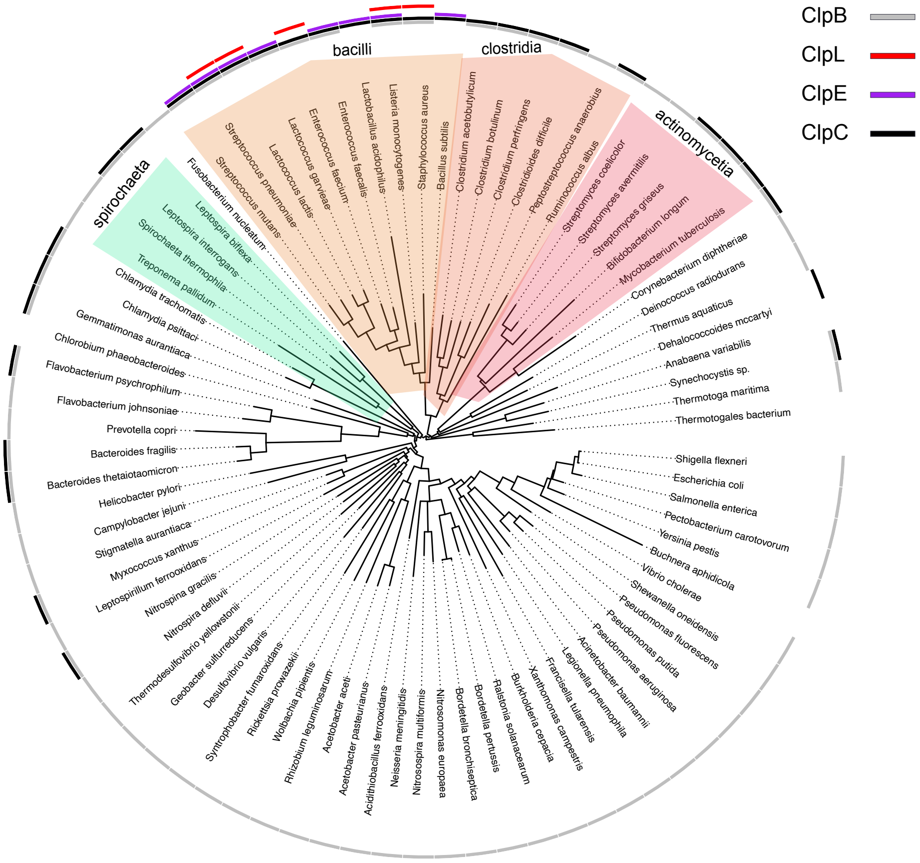

ClpB
ClpL
ClpE
ClpC
spirochaeta
bacilli
clostridia
actinomycetia
Fusobacterium nucleatum
Leptospira biflexa
Leptospira interrogans
Spirochaeta thermophila
Treponema pallidum
Streptococcus mutans
Streptococcus pneumoniae
Lactococcus lactis
Lactococcus garvieae
Enterococcus faecium
Enterococcus faecalis
Lactobacillus acidophilus
Listeria monocytogenes
Staphylococcus aureus
Bacillus subtilis
Clostridium acetobutylicum
Clostridium botulinum
Clostridium perfringens
Clostridioides difficile
Peptostreptococcus anaerobius
Ruminococcus albus
Streptomyces coelicolor
Streptomyces avermitilis
Streptomyces griseus
Bifidobacterium longum
Mycobacterium tuberculosis
Corynebacterium diphtheriae
Deinococcus radiodurans
Thermus aquaticus
Dehalococcoides mccartyi
Anabaena variabilis
Synechocystis sp.
Thermotoga maritima
Thermotogales bacterium
Shigella flexneri
Escherichia coli
Salmonella enterica
Pectobacterium carotovorum
Yersinia pestis
Buchnera aphidicola
Vibrio cholerae
Shewanella oneidensis
Pseudomonas fluorescens
Pseudomonas putida
Pseudomonas aeruginosa
Acinetobacter baumannii
Legionella pneumophila
Francisella tularensis
Xanthomonas campestris
Burkholderia cepacia
Ralstonia solanacearum
Bordetella pertussis
Bordetella bronchiseptica
Nitrosomonas europaea
Nitrosospira multiformis
Neisseria meningitidis
Acidithiobacillus ferrooxidans
Acetobacter pasteurianus
Acetobacter aceti
Rhizobium leguminosarum
Wolbachia pipientis
Rickettsia prowazekii
Syntrophobacter fumaroxidans
Desulfovibrio vulgaris
Geobacter sulfurreducens
Thermodesulfovibrio yellowstonii
Nitrospira defluvii
Nitrospina gracilis
Leptospirillum ferrooxidans
Myxococcus xanthus
Stigmatella aurantiaca
Campylobacter jejuni
Helicobacter pylori
Bacteroides thetaiotaomicron
Bacteroides fragilis
Prevotella copri
Flavobacterium johnsoniae
Flavobacterium psychrophilum
Chlorobium phaeobacteroides
Gemmatimonas aurantiaca
Chlamydia psittaci
Chlamydia trachomatis

Figure S11

Supplementary Figure 12

Mass determination of purified Sa ClpP and Ef ClpP. The purities of Sa ClpP and Ef ClpP were confirmed by intact protein mass determination. Processing of N-terminal residues was observed for Sa ClpP as indicated. The N-terminal methionine residue is indicated in red. *E. coli* ClpP protein (masses: 21,56 or 23,19 kDa: with or without cleavage of the N-terminal propeptide) was not detected, excluding its co-purification.

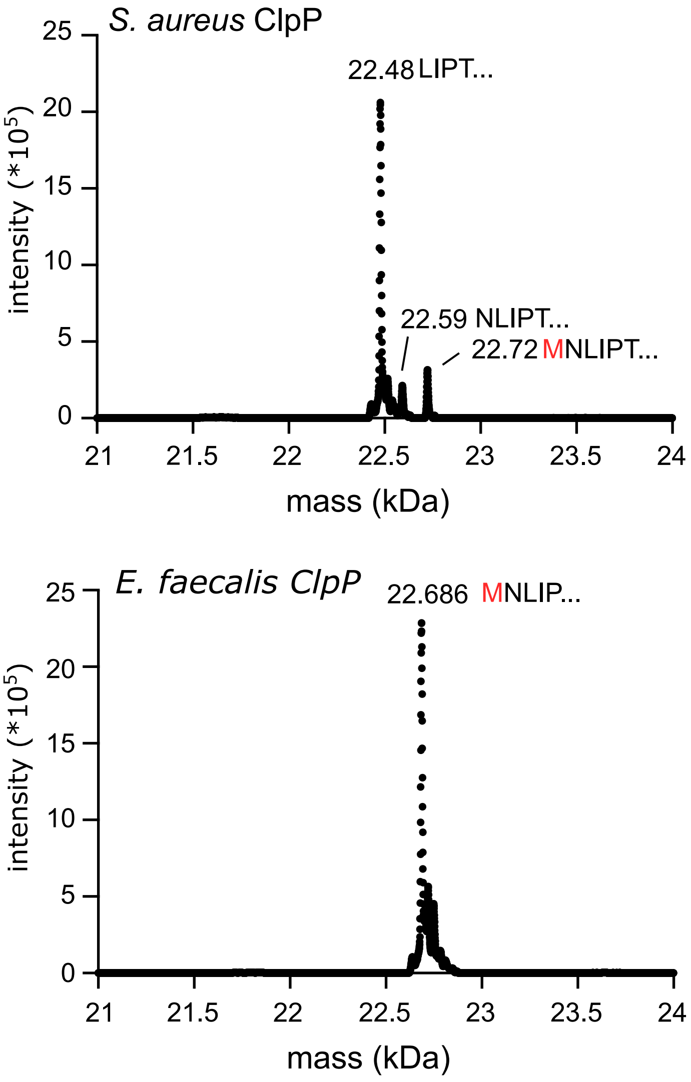
S. aureus ClpP
22.48 LIPT...
22.59 NLIPT...
22.72 MNLIPT...
intensity (*10^5)
25
20
15
10
5
0
21
21.5
22
22.5
23
23.5
24
mass (kDa)
E. faecalis ClpP
22.686 MNLIP...
intensity (*10^5)
25
20
15
10
5
0
21
21.5
22
22.5
23
23.5
24
mass (kDa)

Figure S12
