# Supplementary material for: Structure–function analysis of the bacterial ClpE–ClpP AAA+ protease
Source: J Biol Chem. 2026 Mar 25;302(5):111403. doi: 10.1016/j.jbc.2026.111403 (PMC13125188; doi:10.1016/j.jbc.2026.111403)
Supplement: DeRosa_TableS1 [file mmc2.pdf]

Table S1: Strains and plasmids used in this study

Strain	Description	Source or reference
E. coli XL1 blue	*recA1 endA1 gyrA96 thi-1 hsdR1 supE44 relA1 lac* [F' *proAB lacI^q ΔM15* Tn10 (Tcr)]	Stratagene
E. coli BL21	*F- ompT lon hsdSB gal dcm λ* (DE3)	Novagen
E. coli AG1	recA1 endA1 gyrA96 thi-1 (rK– mK–) supE44 relA1	Agilent
E. coli ΔclpB::kan	MC4100 Δ*clpB*::Km (Kanamycin resistant)	(1)
E.coli ΔclpB	MC4100 Δ*clpB* (Kanamycin sensitive)	This study
**Plasmid**	**Description**	**Source or reference**
pUHE21	Vector control for IPTG-inducible expression in *E. coli ΔclpB::kan* cells; C-terminal His_6-tag has been introduced	This study
pUHE21-*clpE*	Vector for IPTG-inducible expression of *E. faecalis* ClpE (WT and derivatives) in *E. coli ΔclpB::kan* cells	This study
pDS56-*ΔN-clpE*	Vector for IPTG-inducible expression of *E. faecalis* ΔN-ClpE (and derivatives) in *E. coli ΔclpB::kan* cells	
pDS56-*clpP*	Vector for IPTG-inducible expression of *S. aureus* ClpP in *E. coli ΔclpB::kan* cells	(2)
pET24a-*EfclpP*	Vector for IPTG-inducible expression of *E. faecalis* ClpP in *E. coli* BL21 cells	This study
pDS56-*clpC*	Vector for IPTG-inducible expression of *S. aureus* ClpC in *E. coli ΔclpB::kan* cells	(2)
pDS56-*mecA*	Vector for IPTG-inducible expression of MecA in *E. coli ΔclpB::kan* cells	(2)
pET24a-*clpG*	Vector for IPTG-inducible expression of *P. aeruginosa* ClpG in *E. coli* BL21 cells	(1)
pET24a-*mcsA*	Vector for IPTG-inducible expression of *S. aureus* McsA in *E. coli* BL21 cells	This study
pET24a-*mcsB*	Vector for IPTG-inducible expression of *S. aureus* McsB in *E. coli* BL21 cells	This study
pQE32-CtsR	Vector for IPTG-inducible expression of B. subtilis CtsR in *E. coli* AG1 cells	Kursad Turgay lab
pUHE-ClpE-YFP	Vector for IPTG-inducible expression of ClpE-YFP (WT and derivatives) in *E.coli ΔclpB* cells	This study
pDMI.1	Vector harboring *lacI* for tighter repression of IPTG-inducible gene expression in *E. coliΔclpB* cells	(2)
pDMI.1-*clpP*	Vector for IPTG-inducible co-expression of Sa *clpP* in *E. coli ΔclpB* cells	(2)
pDMI.1-*clpP-S98A*	Vector for IPTG-inducible co-expression of Sa *clpP-S98A* in *E. coli ΔclpB* cells	This study

References

1. Katikaridis, P., Romling, U., and Mogk, A. (2021) Basic mechanism of the autonomous ClpG disaggregase *J Biol Chem* 10.1016/j.jbc.2021.100460100460
2. Carroni, M., Franke, K. B., Maurer, M., Jager, J., Hantke, I., Gloge, F. *et al.* (2017) Regulatory coiled-coil domains promote head-to-head assemblies of AAA+ chaperones essential for tunable activity control *eLife* **6**,
