# Supplementary material for: Structure–function analysis of the bacterial ClpE–ClpP AAA+ protease
Source: J Biol Chem. 2026 Mar 25;302(5):111403. doi: 10.1016/j.jbc.2026.111403 (PMC13125188; doi:10.1016/j.jbc.2026.111403)

Table S2: Summary of ATPase and proteolytic activities and oligomerization characteristics of ClpE-WT in comparison to its mutant derivatives.

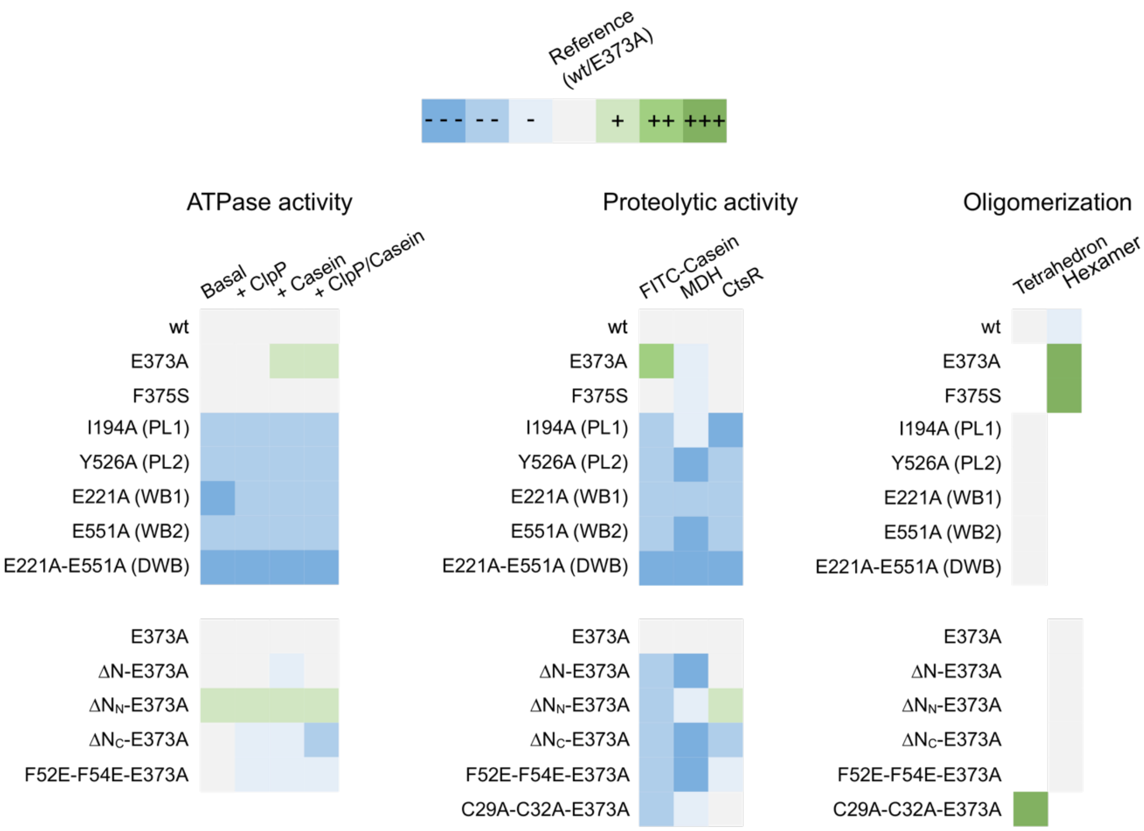

Supplement: De Rosa_Table S2 [file mmc3.pdf]
