# Supplementary material for: Structure–function analysis of the bacterial ClpE–ClpP AAA+ protease
Source: J Biol Chem. 2026 Mar 25;302(5):111403. doi: 10.1016/j.jbc.2026.111403 (PMC13125188; doi:10.1016/j.jbc.2026.111403)
Supplement: DeRosa_Table_S3 [file mmc4.pdf]

Table S3. Cryo-EM data collection, refinement and validation statistics

**Parameter**	**ClpE/ClpP**<br>**(C1) EMDB:** D_1292154861 **PDB:**
Microscope	Titan Krios G3i (TFS)
Detector and energy filter	Gatan K3 + Bioquantum(Ametek)
Nominal magnification (nominal/calibrated at detector)	105k
Voltage (kV)	300
Defocus range (um)	-0.5 to -2.2
Total electron exposure (or fluence, e-/Å2	40
Exposure rate (or flux, e-/pixel/s)	15
Number of frames collected	40
Pixel size (Å)	0.825
Energy filter slit width (eV)	20
Automation software	EPU v3.7
# Micrographs used	14 717
Total # of extracted particles	2 045 530
Total # of refined particles (particles after removing junk)	1 287 964
# of particles in final map	556 680
Resolution of unmasked and masked reconstructions at 0.143 FSC	3.8 / 2.9
Local resolution range (Å)	2.64 to 3.04
Map sharpening B factor (Å2) / (B factor Range)	-86
Model composition<br>Non-hydrogen atoms<br>Protein residues<br>Ligands	 46 205<br>5969<br>ATP:10
B factors ($Å^2$) (min/max/mean)<br>Protein<br>Ligand	 29.92/296.67/59.56<br>41.07/157.99/75.93
Map sharpening EMReady (any)	Yes
Atomic modeling refinement package(s)	Phenix 2.0<br>Coot 0.9.8
CCvolume/CCmask	0.89
Bad bond lengths & bad bond angles	0.003 (0%)<br>0.619 (0%)
Molprobity score	2.44
Clashscore	38.53
Ramachandran plot Z-score	0.98
Ramachandran Plot (%)	Outliers : 0.03 %<br>Allowed : 3.56 %<br>Favored : 96.41 %
Ramachandran rotamers (%)	Outliers : 1.51 %<br>Favored : 70.85 %
CaBLAM outliers (%)	2.7%
EMRinger score	2.86 (unsharpen)/3.32 (EMReady)
